# The Covering Radius and a Discrete Surface Area for Non-Hollow Simplices

**DOI:** 10.1007/s00454-021-00330-3

**Published:** 2021-11-17

**Authors:** Giulia Codenotti, Francisco Santos, Matthias Schymura

**Affiliations:** 1 Institut für Mathematik, Freie Universität Berlin, Arnimallee 2, 14195 Berlin, Germany; 2 Departamento de Matemáticas, Estadística y Computación, Universidad de Cantabria, Av. de Los Castros 48, 39005 Santander, Spain; 3 Institut für Mathematik, BTU Cottbus-Senftenberg, Platz der Deutschen Einheit 1, 03046 Cottbus, Germany

**Keywords:** Covering radius, Discrete surface area, Lattice polytopes, Volume, 52B20, 11H31, 11H06, 52A38

## Abstract

We explore upper bounds on the covering radius of non-hollow lattice polytopes. In particular, we conjecture a general upper bound of *d*/2 in dimension *d*, achieved by the “standard terminal simplices” and direct sums of them. We prove this conjecture up to dimension three and show it to be equivalent to the conjecture of González-Merino and Schymura (Discrete Comput. Geom. **58**(3), 663–685 (2017)) that the *d*-th covering minimum of the standard terminal *n*-simplex equals *d*/2, for every $n\ge d$. We also show that these two conjectures would follow from a discrete analog for lattice simplices of Hadwiger’s formula bounding the covering radius of a convex body in terms of the ratio of surface area versus volume. To this end, we introduce a new notion of discrete surface area of non-hollow simplices. We prove our discrete analog in dimension two and give strong evidence for its validity in arbitrary dimension.

## Introduction

The *covering radius* of a convex body *K* in ${\mathbb {R}}^d$ with respect to a lattice $\Lambda$ is defined as

$$\begin{aligned} \mu (K,\Lambda ) = \min {\{\mu \ge 0 : \mu K + \Lambda ={\mathbb {R}}^d\}}. \end{aligned}$$

For us, a *lattice* is always a full-dimensional linear image of ${\mathbb {Z}}^d$. Unless stated otherwise, we consider $\Lambda ={\mathbb {Z}}^d$ and just write $\mu (K)$. A convex body *K* is called *hollow* or *lattice-free* (with respect to $\Lambda$) if ${{\,\mathrm{int}\,}}(K)\cap \Lambda =\emptyset$, where ${{\,\mathrm{int}\,}}(K)$ denotes the interior of *K*. With this notion, the covering radius $\mu (K,\Lambda )$ can be equivalently described as the greatest $\mu \ge 0$ such that the dilation $\mu K$ admits a hollow translate.

The covering radius is a classical parameter in the Geometry of Numbers, in particular in the realm of transference results, the reduction of quadratic forms, and Diophantine Approximations (cf. [12] for some background). In the context of the so-called *flatness theorem* it also proved crucial in Lenstra’s landmark paper [19] on solving Linear Integer Programming in fixed dimension in polynomial time (see [17] for more on the flatness theorem). More recent applications of the covering radius include (a) the classification of lattice polytopes in small dimensions (see [15] and references therein), (b) distances between optimal solutions of mixed-integer programs and their linear relaxations [23], (c) unique-lifting properties of maximal lattice-free polyhedra [1], and (d) another viewpoint on the famous Lonely Runner Problem [14].

The covering radius is clearly invariant under translations of the body *K*, and for every invertible matrix $A\in {\mathbb {R}}^{d\times d}$, we have $\mu (AK,A\Lambda )=\mu (K,\Lambda )$. Hence, the covering radius is invariant under *unimodular transformations*, which are affine maps $x\mapsto Ux+z$, where $z\in {\mathbb {Z}}^d$ and $U\in {{\,\mathrm{GL}\,}}_d({\mathbb {Z}})$ is a *unimodular matrix*. The behavior with respect to inclusions is as follows: For convex bodies $K\subseteq K'$ and lattices $\Lambda '\subseteq \Lambda$, we have $\mu (K',\Lambda )\le \mu (K,\Lambda )\le \mu (K,\Lambda ')$.

We are interested in upper bounds on the covering radius of non-hollow *lattice polytopes*, that is, polytopes all of whose vertices are lattice points. If we drop the non-hollow condition, the maximum covering radius of a lattice *d*-polytope equals *d*. This follows since every lattice *d*-polytope contains a lattice *d*-simplex and for lattice simplices the bound is readily obtained (cf. [10, (19)]). Moreover, equality holds if and only if the lattice polytope is a *unimodular simplex*; that is, one of the form ${{\,\mathrm{conv}\,}}{\{{\mathbf {0}},b_1,\ldots ,b_d\}}$, where $\{b_1,\ldots ,b_d\}$ is a lattice basis for $\Lambda$, or a lattice translate of that (see Corollary 4.13 for a proof of a more general statement).

The existence of interior lattice points makes the problem more difficult and interesting. The natural candidate to play the role of the unimodular simplex is

$$\begin{aligned} S({\mathbf {1}}_{d+1}):={{\,\mathrm{conv}\,}}{\{{-}{\mathbf {1}}_d,e_1,\ldots ,e_d\}}, \end{aligned}$$

since it is the unique non-hollow lattice *d*-polytope of minimum volume (see [4, Thm. 1.2]). Here ${\mathbf {1}}_d = (1,\ldots ,1)$ denotes the all-one vector in dimension *d*, and $e_i$ denotes the *i*th coordinate unit vector.^1^

The covering radius of $S({\mathbf {1}}_{d+1})$ was computed in [10, Prop. 4.9]:

1 $$\begin{aligned} \mu (S({\mathbf {1}}_{d+1}),{\mathbb {Z}}^d) = \frac{d}{2}. \end{aligned}$$

Since the covering radius is additive with respect to direct sums (see Sect. 2.1), direct sums of simplices of the form $S({\mathbf {1}}_l)$ or lattice translates thereof also have covering radius equal to *d*/2. We conjecture that this procedure gives *all* the non-hollow lattice polytopes of maximum covering radius in a given dimension:

### Conjecture A

Let $P \subseteq {\mathbb {R}}^d$ be a non-hollow lattice *d*-polytope. Then

$$\begin{aligned} \mu (P) \le \frac{d}{2}, \end{aligned}$$

with equality if and only if *P* is obtained by direct sums and/or translations of simplices of the form $S({\mathbf {1}}_l)$.

### Example 1.1

In dimension two, $S({\mathbf {1}}_3)$ has covering radius 1, and so do the following triangle and square:

$$\begin{aligned} S({\mathbf {1}}_{2})\oplus ((1+ S({\mathbf {1}}_{2}))&= {{\,\mathrm{conv}\,}}{\{(1,0), (-1,0), (0,2)\}},\\ S({\mathbf {1}}_{2})\oplus S({\mathbf {1}}_{2})&= {{\,\mathrm{conv}\,}}{\{(1,0), (-1.0), (0,1), (0, -1)\}}. \end{aligned}$$

In dimension three, translations and/or direct sums of the $S({\mathbf {1}}_l)$s produce nine pairwise non-equivalent non-hollow lattice 3-polytopes of covering radius 3/2, that we describe in Lemma 3.83.8.

One motivation for Conjecture A is as follows. The *d*-*th covering minimum* of a convex body $K \subseteq {\mathbb {R}}^n$ with respect to a lattice $\Lambda \subseteq {\mathbb {R}}^n$ is defined as

$$\begin{aligned} \mu _d(K, \Lambda ) := \max _{\pi } \mu (\pi (K),\pi (\Lambda )), \end{aligned}$$

where $\pi$ runs over all linear projections $\pi :{\mathbb {R}}^n\rightarrow {\mathbb {R}}^d$ such that $\pi (\Lambda )$ is a lattice. Covering minima were introduced by Kannan and Lovász [17] and interpolate between $\mu _n(K)=\mu (K)$ and $\mu _1(K)$, the reciprocal of the *lattice width* of *K*.

Since $S({\mathbf {1}}_{n+1})$ projects to $S({\mathbf {1}}_{d+1})$ for every $d < n$, we use (1) and get

2 $$\begin{aligned} \mu _d(S({\mathbf {1}}_{n+1})) \ge \mu _d(S({\mathbf {1}}_{d+1})) = \frac{d}{2}. \end{aligned}$$

The converse inequality was conjectured in [10]:

### Conjecture B

([10, Rem. 4.10])  For every $n \in {\mathbb {N}}$ and $d \le n$,

3 $$\begin{aligned} \mu _d(S({\mathbf {1}}_{n+1}))= \frac{d}{2}. \end{aligned}$$

In Sect. 3 we prove:

### Theorem 1.2

(equivalence of Conjectures A and B, Sect. 3.1)  For each $d\in {\mathbb {N}}$, the following are equivalent:

(i) $\mu (P)\le \ell /2$ for every non-hollow lattice $\ell$-polytope *P* and for every $\ell \le d$.
(ii) Conjecture B holds for every $\ell \le d$. That is, $\mu _\ell (S({\mathbf {1}}_{n+1}))=\ell /2$, for every $\ell ,n\in {\mathbb {N}}$ with $\ell \le d\le n$.

### Theorem 1.3

(Corollary 3.6 and Theorem 3.13)  *Conjecture* A, hence also *Conjecture* B, holds in dimension up to three.

The computation of the covering radius for $S({\mathbf {1}}_{d+1})$ can be generalized to the following class of simplices: For each $\omega =(\omega _0,\dots ,\omega _d) \in {\mathbb {R}}_{> 0}^{d+1}$, we define

$$\begin{aligned} S(\omega ) := {{\,\mathrm{conv}\,}}{\{ -\omega _0 {\mathbf {1}}_d, \omega _1 e_1, \ldots , \omega _d e_d\}}. \end{aligned}$$

In Sect. 5 we derive the following closed formula for $\mu (S(\omega ))$. Therein and in the rest of the paper we denote by ${{\,\mathrm{Vol}\,}}_\Lambda K$ the *normalized volume* of a convex body *K* with respect to a lattice $\Lambda$, which equals the Euclidean volume ${{\,\mathrm{vol}\,}}K$ of *K* normalized so that a unimodular simplex of $\Lambda$ has volume one.

### Theorem 1.4

(Sect. 5.1)  For every $\omega \in {\mathbb {R}}^{d+1}_{>0}$, we have

$$\begin{aligned} \mu (S(\omega ))=\frac{\sum _{0\le i<j\le d}{1}/({\omega _i\omega _j})}{\sum _{i=0}^d{1}/{\omega _i}}=\frac{1}{2}\cdot \frac{\sum _{i=0}^d \mathrm {Vol}_{\pi _i({\mathbb {Z}}^d)}(\pi _i(S(\omega )))}{{{\,\mathrm{Vol}\,}}_{{\mathbb {Z}}^d}(S(\omega ))}, \end{aligned}$$

where $\pi _i:{\mathbb {R}}^d\rightarrow {\mathbb {R}}^{d-1}$ is the linear orthogonal projection along the line through the origin and the *i*-th vertex of $S(\omega )$.

In [10], the authors conjecture an optimal lower bound on the *covering product* $\mu _1(K)\cdot \ldots \cdot \mu _d(K)\cdot {{\,\mathrm{Vol}\,}}_{{\mathbb {Z}}^d}K$ for any convex body $K \subseteq {\mathbb {R}}^d$. As a consequence of the explicit formula for $\mu (S(\omega ))$, we confirm this conjecture for the simplices $S(\omega )$ (see Corollary 5.2).

Observe that the volume expression on the right in Theorem 1.4 can be defined for every simplex with the origin in its interior as follows:

### Definition 1.5

Let $S={{\,\mathrm{conv}\,}}{\{v_0,\ldots ,v_d\}}$ be a *d*-simplex with the origin in its interior. We say that *S* has *rational vertex directions* if the line through the origin and the vertex $v_i$ has rational direction, for every $0\le i\le d$. Writing $\pi _i:{\mathbb {R}}^d \rightarrow {\mathbb {R}}^{d-1}$ for a linear projection vanishing at $v_i$, we define the *discrete surface area* of such a simplex *S* as

$$\begin{aligned} {{\,\mathrm {Surf}\,}}_{{\mathbb {Z}}^d}(S) := \sum _{i=0}^d {{\,\mathrm {Vol}\,}}_{\pi _i({\mathbb {Z}}^d)}(\pi _i(S)). \end{aligned}$$

Note that ${{\,\mathrm{Vol}\,}}_{\pi _i({\mathbb {Z}}^d)} \left( \pi _i(S)\right) = {{\,\mathrm {Vol}\,}}_{\pi _i({\mathbb {Z}}^d)}\left( \pi _i(F_i)\right)$, with $F_i$ being the facet of *S* opposite to the vertex $v_i$. In this sense, the sum of these numbers is indeed a version of the “surface area” of *S*, except that the volume of each facet is computed with respect to the lattice projected from the opposite vertex. Motivated by this definition and Theorem 1.4 we propose the following conjecture, which is the main object of study in this paper:

### Conjecture C

Let *S* be a *d*-simplex with the origin in its interior and with rational vertex directions. Then

4 $$\begin{aligned} \mu (S) \le \frac{1}{2}\cdot \frac{{{\,\mathrm{Surf}\,}}_{{\mathbb {Z}}^d}S}{{{\,\mathrm{Vol}\,}}_{{\mathbb {Z}}^d}S}. \end{aligned}$$

We formulate this conjecture only for simplices *S* rather than for arbitrary polytopes that contain the origin and have rational vertex directions, because without further study it is not clear how the discrete surface area ${{\,\mathrm{Surf}\,}}_{{\mathbb {Z}}^d}S$ can be extended in a meaningful way. For example, we could project along the vertex directions as in the simplex case, but then the correspondence with the opposite facet is lost.

In Sect. 4 we give additional motivation for Conjecture C. We show that it implies Conjecture A (Corollary 4.3), that it holds in dimension two (Theorem 4.9), and that in arbitrary dimension it holds up to a factor of two (Proposition 4.4).

Covering criteria such as the one in Conjecture C are rare in the literature, but very useful as they reduce the question of covering to computing less complex geometric functionals such as volume or (variants of the) surface area (cf. [11, Sect. 31]). A classical inequality of this type is the following result of Hadwiger. We regard Conjecture C as a discrete analog thereof.

### Theorem 1.6

([13])  For every convex body *K* in ${\mathbb {R}}^d$,

$$\begin{aligned} \mu (K) \le \frac{1}{2}\cdot \frac{{{\,\mathrm{surf}\,}}K}{{{\,\mathrm{vol}\,}}K}, \end{aligned}$$

where ${{\,\mathrm{vol}\,}}K$ and ${{\,\mathrm{surf}\,}}K$ are the Euclidean volume and surface area of *K*.

Observe that the statement of Conjecture C is more intrinsic than Hadwiger’s inequality. This is because the Euclidean surface area is not invariant under unimodular transformations, so that the bound in Theorem 1.6 depends on the particular representative of *K* in its unimodular class. Moreover, the inequality only holds for the standard lattice ${\mathbb {Z}}^d$ and cannot easily be transfered to other lattices (cf. [24] for partial results for arbitrary lattices). In contrast, our proposed relation in Conjecture C*is* unimodularly invariant and there is no loss of generality in restricting to the standard lattice as we do (see Lemma 4.2 for details on these claims). Moreover, our proposed inequality in Conjecture C is tight for the large class of simplices $S(\omega )$. In Sect. 4.4, we complement our investigations on Conjecture C by extending it to the case where the origin lies in the boundary of the simplex *S*, rather than in the interior.

Another way to extend Conjecture A is to ask for the maximal covering radius among lattice polytopes with at least $k\ge 1$ interior lattice points. The natural conjecture is:

### Conjecture D

Let $k,d\in {\mathbb {N}}$ be nonnegative integers. Then for every lattice *d*-polytope *P* with *k* interior lattice points we have

$$\begin{aligned} \mu (P) \le \frac{d-1}{2} + \frac{1}{k+1}. \end{aligned}$$

Equality holds for $k=1$ if and only if *P* is obtained by direct sums and/or translations of simplices of the form $S({\mathbf {1}}_l)$, and for $k\ge 2$, if and only if *P* is obtained by direct sums and/or translations of the segment $[0,k+1]$ and simplices $S({\mathbf {1}}_l)$.

In Sect. 6 we prove this conjecture in dimension two (see Theorem 6.3). Observe that no analog of Conjecture D makes sense for other covering minima. Indeed, the maximum *d*-th covering minimum $\mu _d$ among non-hollow lattice *n*-polytopes with *k* interior lattice points does not depend on *k* or *n*, for $d<n$: It equals the maximum covering radius among non-hollow lattice *d*-polytopes, since every non-hollow lattice *d*-polytope can be obtained as the projection of a $(d+1)$-polytope with arbitrarily many interior lattice points. In fact, assuming Conjecture A this maximum is given by

$$\begin{aligned} \mu _d(S(k,1,\ldots ,1)) = \mu _d(S({\mathbf {1}}_{d+1})) = \frac{d}{2}, \qquad \text {for all } n>d \text { and } k\in {\mathbb {N}}. \end{aligned}$$

Summing up, the relationship between our conjectures is as follows:

$$\begin{aligned} \begin{array}{cccc} &{}&{}Conjecture ~\mathrm{C}\\ &{}&{}\Downarrow \\ Conjecture ~\mathrm{D}~ &{} \Rightarrow &{} Conjecture ~\mathrm{A} \\ &{}&{}\Downarrow \\ &{}&{}Conjecture ~\mathrm{A}&{}\text { without equality case}\\ &{}&{}\Updownarrow \\ &{}&{}Conjecture ~\mathrm{B}\\ \end{array} \end{aligned}$$

A summary of our results is that all these conjectures hold in dimension two, that Conjecture A holds in dimension three, and that Conjecture C holds for the simplices of the form $S(\omega )$.

## Preliminaries

This section develops some tools that will be essential for our analyses. We first describe how the covering radius behaves with respect to projections, and more importantly, that it is an additive functional on direct sums of convex bodies and lattices. Afterwards we introduce and study the concept of *tight covering* which facilitates our characterizations of equality, for example the one in Theorem 1.3.

### Projection and Direct Sum

#### Lemma 2.1

Let $K \subseteq {\mathbb {R}}^d$ be a convex body containing the origin, and let $\pi :{\mathbb {R}}^d \rightarrow {\mathbb {R}}^l$ be a rational linear projection, so that $\pi ({\mathbb {Z}}^d)$ is a lattice. Let $Q=K \cap \pi ^{-1}({\mathbf {0}})$ and let $L= \pi ^{-1}({\mathbf {0}})$ be the linear subspace spanned by *Q*. Then we have

$$\begin{aligned} \mu (K,{\mathbb {Z}}^d) \le \mu (Q,{\mathbb {Z}}^d \cap L) + \mu (\pi (K),\pi ({\mathbb {Z}}^d)). \end{aligned}$$

#### Proof

Let us abbreviate $\mu _Q=\mu (Q,{\mathbb {Z}}^d\cap L)$ and $\mu _\pi =\mu (\pi (K),\pi ({\mathbb {Z}}^d))$. Let $x\in {\mathbb {R}}^d$ be arbitrary. Then, $\pi (x)$ is covered by $\mu _\pi \cdot \pi (K)+\pi ({\mathbb {Z}}^d)=\pi (\mu _\pi K+{\mathbb {Z}}^d)$. Hence, there exists a point $x'\in {\mathbb {R}}^d$ such that the segment $[x,x']$ is parallel to *L* and such that $x'$ is covered by $\mu _\pi K+{\mathbb {Z}}^d$. On the other hand, $y=x-x'\in L$ is covered by $\mu _QQ+({\mathbb {Z}}^d\cap L)$. Since $Q\subseteq K$, this implies that $x=y+x'$ is covered by $(\mu _Q+\mu _{\pi }) K+{\mathbb {Z}}^d$, as claimed. $\square$

A particularly interesting case of the above result is when *K* decomposes as a direct sum. Let ${\mathbb {R}}^d=V\oplus W$ be a decomposition into complementary linear subspaces with $\dim V=\ell$ and $\dim W=d-\ell$. The *direct sum* of two convex bodies $K\subseteq V$, $L\subseteq W$ both containing the origin is defined as

$$\begin{aligned} K\oplus L :=\{\lambda x + (1-\lambda ) y :x \in K,\,y \in L, \,\lambda \in [0,1]\} \subseteq {\mathbb {R}}^d. \end{aligned}$$

The direct sum of two lattices $\Lambda \subseteq V$, $\Gamma \subseteq W$ is defined as

$$\begin{aligned} \Lambda \oplus \Gamma :=\{ x + y : x \in \Lambda ,\,y \in \Gamma \} \subseteq {\mathbb {R}}^d. \end{aligned}$$

With these definitions we can now formulate

#### Corollary 2.2

Let ${\mathbb {R}}^d = V \oplus W$ be a decomposition as above, let $K \subseteq V$, $L \subseteq W$ be convex bodies containing the origin, and let $\Lambda \subseteq V$, $\Gamma \subseteq W$ be lattices. Then

$$\begin{aligned} \mu _d(K \oplus L, \Lambda \oplus \Gamma ) = \mu _\ell (K, \Lambda ) + \mu _{d-\ell }(L, \Gamma ). \end{aligned}$$

#### Proof

The inequality $\mu _d(K\oplus L,\Lambda \oplus \Gamma )\le \mu _\ell (K,\Lambda )+\mu _{d-\ell }(L,\Gamma )$ is a special case of Lemma 2.1, via the natural projection ${\mathbb {R}}^d=V\oplus W \rightarrow V$.

For the other inequality, let $x\in V$ be a point not covered by $cK+\Lambda$ for some $c<\mu _\ell (K,\Lambda )$ and let $y\in W$ be a point not covered by ${\bar{c}}L+\Gamma$ for some ${\bar{c}}<\mu _{d-\ell }(L,\Gamma )$. We claim that $x+y\in V\oplus W={\mathbb {R}}^d$ is not covered by $(c+{\bar{c}})(K\oplus L)+\Lambda \oplus \Gamma$, and thus $c+{\bar{c}}\le \mu _d(K\oplus L,\Lambda \oplus \Gamma )$. Since *c* and ${\bar{c}}$ were taken arbitrarily, this implies $\mu _\ell (K,\Lambda ) + \mu _{d-\ell }(L, \Gamma ) \le \mu _d(K \oplus L, \Lambda \oplus \Gamma )$.

Assume, to the contrary, that $x+y\in (c+{\bar{c}})(K \oplus L)+\Lambda \oplus \Gamma$, that is, $x+y=(c+{\bar{c}})(\lambda p +(1-\lambda ) q) + w + z$, for some $\lambda \in [0,1]$, $p \in K$, $q\in L$, $w\in \Lambda$, and $z\in \Gamma$. Since the sums are direct, we get $x=(c+{\bar{c}})\lambda p+w$ and $y=(c+{\bar{c}})(1-\lambda ) q+z$, which by assumption implies $(c+{\bar{c}})\lambda >c$ and $(c+{\bar{c}})(1-\lambda )>{\bar{c}}$. These two inequalities cannot hold at the same time, and we arrive at a contradiction. $\square$

### Tight Covering

#### Definition 2.3

Let $K\subseteq {\mathbb {R}}^d$ be a convex body and let $\Lambda$ be a lattice. Then, *K* is called *tight for* $\Lambda$ if for every convex body $K'\supsetneq K$, we have

$$\begin{aligned} \mu (K',\Lambda ) < \mu (K,\Lambda ). \end{aligned}$$

#### Definition 2.4

Let $K\subseteq {\mathbb {R}}^d$ be a convex body of covering radius $\mu$ with respect to a lattice $\Lambda$. A point $p\in {\mathbb {R}}^d$ is *last covered by* *K* if

$$\begin{aligned} p\notin {{\,\mathrm{int}\,}}(\mu \cdot K) + \Lambda . \end{aligned}$$

Let *P* be a *d*-polytope, let *F* be a facet of *P*, and let *p* be a point that is last covered by *P*. We say that *p* *needs* *F* if $p\in {{\,\mathrm{relint}\,}}(\mu \cdot F)+\Lambda$.

#### Lemma 2.5

Let $K \subseteq {\mathbb {R}}^d$ be a convex body of covering radius $\mu$ with respect to a lattice $\Lambda$. Then, the following properties are equivalent:

(i) *K* is tight for $\Lambda$.
(ii) *K* is a polytope and for every facet *F* of *K* and for every last covered point *p*, *p* needs *F*.
(iii) *K* is a polytope and every facet of every hollow translate of $\mu \cdot K$ is non-hollow.
(iv) Every hollow translate of $\mu \cdot K$ is a maximal hollow convex body with respect to inclusion.

#### Proof

The equivalence of (iii) and (iv) is the characterization of maximal hollow convex bodies by Lovász [20]. For the equivalence of (i) and (iv) observe that, by definition, $\mu$ is the largest constant such that (a) $\mu \cdot K$ has a hollow lattice translate and (b) the inequality $\mu (K',\Lambda )<\mu (K,\Lambda )$ in the definition of tightness is nothing but maximality of all such hollow translates.

We now show the equivalence of (i) and (ii). Suppose there is a facet *F* of *K* that is not needed by some last covered point *p*. Let $K'={{\,\mathrm{conv}\,}}{(K\cup \{x\})}$, where $x\notin K$ is a point *beyond* *F*, meaning that *x* violates the inequality that defines *F*, but satisfies all other facet-inducing inequalities of *K*. Then

$$\begin{aligned} \mu (K',\Lambda ) = \mu (K,\Lambda ) \end{aligned}$$

because *p* is still a last covered point of $K'$ (for the same dilate $\mu$).

Conversely, if *K* is not tight let $K'$ be a convex body strictly containing *K* and that has the same covering radius. Let *F* be a facet of *K* with ${{\,\mathrm{relint}\,}}F\subseteq {{\,\mathrm{int}\,}}K'$. Let *p* be a point that is last covered by $K'$. Since the covering radii are equal and $K \subsetneq K'$, *p* must also be last covered by *K*. Since we chose *F* so that ${{\,\mathrm{relint}\,}}F$ is in the interior of $K'$, *p* does not need *F*. $\square$

#### Example 2.6

It is not sufficient for tightness that “every facet is needed by *some* last covered point.” An example showing this is the hexagon $P={{\,\mathrm{conv}\,}}\{\pm (1,0)$, $\pm (0,1)$, $\pm (1,1)\}$ with respect to the integer lattice. *P* has covering radius 2/3, the same as the triangle ${{\,\mathrm{conv}\,}}{\{(-1,1),(2,1),(-1,-2)\}}$ that properly contains it, so it is not tight. It has two orbits of last covered points, with representatives ${\pm }(2/3,1/3)$, each of which needs three of the six edges of *P*.

#### Lemma 2.7

Every simplex is tight for every lattice.

#### Proof

We use Lemma 2.5. Let $\Delta$ be a simplex of covering radius $\mu$ with respect to a lattice $\Lambda$, and let *p* be a point last covered by $\Delta$. That is, $p \notin {{\,\mathrm{int}\,}}(\mu \Delta ) + \Lambda$. Let $F_0,F_1,\ldots ,F_d$ be the facets of $\Delta$, with interior facet normals $v_0,\dots ,v_d$.

Every neighborhood of *p* is covered by $\mu \Delta + \Lambda$, and *p* can only lie in lattice translates of the boundary of $\mu \Delta$. Suppose, in order to get a contradiction, that a certain facet $F_i$ is not needed by *p*. This implies that for every $\mu \Delta + z$ ($z\in \Lambda$) containing *p* there is a facet $F_j\ne F_i$ such that $\mu \Delta + z \subset H_j^p$, where

$$\begin{aligned} H_j^p:=\{x \in {\mathbb {R}}^d : v_j^\intercal x \le v_j^\intercal p\} \end{aligned}$$

is the translation to *p* of the *j*-th facet-defining half-space of $\Delta$. This implies that we have a neighborhood of *p* covered by the *d* affine half-spaces with *p* in the boundary corresponding to the indices ${j\ne i}$. This is impossible since the corresponding *d* normals are linearly independent. $\square$

#### Lemma 2.8

Let $K_1$ and $K_2$ be convex bodies containing the origin and let $\Lambda _1$ and $\Lambda _2$ be lattices. Then, $K_1$ and $K_2$ are tight for $\Lambda _1$ and $\Lambda _2$, respectively, if and only if $K_1 \oplus K_2$ is tight for $\Lambda _1 \oplus \Lambda _2$.

#### Proof

First of all, let $K'\supsetneq K_1\oplus K_2$ be a convex body and let $K'_1$ and $K'_2$ be the projection of $K'$ onto the linear span of $K_1$ and $K_2$, respectively. Clearly, either $K'_1\supsetneq K_1$ or $K'_2\supsetneq K_2$, so that by Corollary 2.2 and the tightness of $K_1$ and $K_2$, we have

$$\begin{aligned} \mu (K_1 \oplus K_2, \Lambda _1 \oplus \Lambda _2)&= \mu (K_1,\Lambda _1) + \mu (K_2,\Lambda _2) > \mu (K'_1,\Lambda _1) + \mu (K'_2,\Lambda _2) \\&= \mu (K'_1 \oplus K'_2, \Lambda _1 \oplus \Lambda _2) \ge \mu (K', \Lambda _1 \oplus \Lambda _2), \end{aligned}$$

since $K'_1 \oplus K'_2 \subseteq K'$. Therefore, $K_1 \oplus K_2$ is tight for $\Lambda _1 \oplus \Lambda _2$.

Conversely, if say $K_1$ is not tight for $\Lambda _1$, then there exists $K'_1 \supsetneq K_1$ such that $\mu (K_1,\Lambda _1) = \mu (K'_1,\Lambda _1)$. Then, $K'_1 \oplus K_2 \supsetneq K_1 \oplus K_2$ and by Corollary 2.2,

$$\begin{aligned} \mu (K'_1 \oplus K_2,\Lambda _1 \oplus \Lambda _2)&= \mu (K'_1,\Lambda _1) + \mu (K_2,\Lambda _2)\\&= \mu (K_1,\Lambda _1) + \mu (K_2,\Lambda _2) = \mu (K_1 \oplus K_2,\Lambda _1 \oplus \Lambda _2), \end{aligned}$$

so $K_1 \oplus K_2$ is not tight for $\Lambda _1 \oplus \Lambda _2$. $\square$

#### Lemma 2.9

Let $\Lambda ' \subsetneq \Lambda$ be two lattices in ${\mathbb {R}}^d$, and let $K \subseteq {\mathbb {R}}^d$ be a convex body. Then

$$\begin{aligned} \mu (K, \Lambda ) \le \mu (K, \Lambda '). \end{aligned}$$

#### Proof

Let $\mu = \mu (K, \Lambda )$ and $\mu ' = \mu (K, \Lambda ')$. Then, $\mu ' K + \Lambda ' \subseteq \mu ' K + \Lambda$, so $\mu \le \mu '$. $\square$

#### Remark 2.10

(i) An example where equality holds in Lemma 2.9 is the following: Let $K= [-1,1]^d$ and let $\Lambda$ be an arbitrary refinement of ${\mathbb {Z}}^d$ contained in ${\mathbb {R}}^{d-1} \times {\mathbb {Z}}$. Then, $\mu (K,{\mathbb {Z}}^d) = \mu (K,\Lambda )=1/2$.
(ii) The inequality in Lemma 2.9 may not be strict, even for simplices. An example is the simplex $(I\oplus I')' \oplus I$ of Lemma 3.83.8 below. It has the same covering radius as $S({\mathbf {1}}_4)$ (equal to 3/2), yet it is isomorphic to $S({\mathbf {1}}_4)$ when regarded with respect to the sublattice of index two generated by its vertices and its interior lattice point. This can easily be derived from its depiction in the bottom-center of Fig. 2, or from its coordinates in Table 1 (in these coordinates the sublattice is $\{(x,y,z)\in {\mathbb {Z}}^3: x\in 2{\mathbb {Z}}\}$).

## Conjectures A and B: Equivalence and Small Dimensions

### Equivalence of Conjectures A and B

As an auxiliary result we first reduce Conjecture A to lattice simplices.

#### Lemma 3.1

Every non-hollow lattice polytope contains a non-hollow lattice simplex of possibly smaller dimension.

#### Proof

Let *P* be a non-hollow lattice polytope and $p\in {{\,\mathrm{int}\,}}(P)\cap {\mathbb {Z}}^d$. Applying Carathéodory’s Theorem to an expression of *p* as a convex combination of the vertex set of *P*, we obtain an affinely independent subset of vertices that still has *p* as a convex combination. The vertices involved in that convex combination form a non-hollow simplex contained in *P*. $\square$

#### Corollary 3.2

Conjecture A reduces to lattice simplices. More precisely, Conjecture A holds in every dimension $\le d$ if and only if it holds for lattice simplices in every dimension $\le d$.

#### Proof

One direction is trivially true. We prove the other one by induction on *d*. Let $P\subseteq {\mathbb {R}}^d$ be a non-hollow lattice polytope. In view of Lemma 3.1, we find an $\ell$-dimensional non-hollow lattice simplex $S\subseteq P$. If $\ell =d$, then we simply have $\mu (P) \le \mu (S)$. So, let us assume that $\ell <d$ and assume that Conjecture A is proven for any dimension $<d$. Assume also that *S* contains the origin in its interior and write $L_S$ for the linear hull of *S*. We now apply Lemma 2.1 to the projection $\pi$ onto $L_S^\perp$. Observe that $S\subseteq P\cap \pi ^{-1}({\mathbf {0}})=P\cap L_S$, and that *S* is non-hollow with respect to ${\mathbb {Z}}^d \cap L_S$ and $\pi (P)$ is non-hollow with respect to the lattice $\pi ({\mathbb {Z}}^d)$. We get that

$$\begin{aligned} \qquad \qquad \mu (P) \le \mu (S,{\mathbb {Z}}^d \cap L_S) + \mu (\pi (P),\pi ({\mathbb {Z}}^d)) \le \frac{\ell }{2} + \frac{d-\ell }{2} = \frac{d}{2}. \qquad \qquad \square \end{aligned}$$

#### Proof of Theorem 1.2

Suppose first that for $\ell \le d$ every lattice $\ell$-polytope *P* has $\mu (P)\le \ell /2$. Since $S({\mathbf {1}}_{n+1})$ projects to $S({\mathbf {1}}_{\ell +1})$, we have by (1),

$$\begin{aligned} \mu _\ell (S({\mathbf {1}}_{n+1}),{\mathbb {Z}}^n) \ge \mu _\ell (S({\mathbf {1}}_{\ell +1}),{\mathbb {Z}}^\ell ) = \frac{\ell }{2}. \end{aligned}$$

For the converse inequality, let $\pi :{\mathbb {R}}^n\rightarrow {\mathbb {R}}^\ell$ be an integer projection along which the value of $\mu _\ell (S({\mathbf {1}}_{n+1}))$ is attained. Then, $\pi (S({\mathbf {1}}_{n+1}))$ is non-hollow with respect to the lattice $\pi ({\mathbb {Z}}^n)$, and thus

$$\begin{aligned} \mu _\ell (S({\mathbf {1}}_{n+1}),{\mathbb {Z}}^n) = \mu _\ell (\pi (S({\mathbf {1}}_{n+1})), \pi ({\mathbb {Z}}^n)) \le \frac{\ell }{2}. \end{aligned}$$

For the reverse implication (ii) $\Rightarrow$ (i), suppose Conjecture B holds in every dimension $\ell \le d$. Let *P* be a lattice $\ell$-polytope with at least one interior lattice point, which without loss of generality we assume to be the origin ${\mathbf {0}}$. By Corollary 3.2 we can assume *P* to be a simplex, and we let $v_0,\dots ,v_\ell$ be its vertices. Let $(b_0,\ldots ,b_\ell ) \in {\mathbb {N}}^{\ell +1}$ be a tuple of the barycentric coordinates of ${\mathbf {0}}$ in *P*; that is, assume that

5 $$\begin{aligned} {\mathbf {0}}= \frac{1}{N} \sum _{i=0}^\ell b_i v_i, \end{aligned}$$

where $N=\sum _{i=0}^\ell b_i\ge \ell +1$. Consider the $(N-1)$-dimensional simplex $S({\mathbf {1}}_{N})$, and the affine projection $\pi :{\mathbb {R}}^{N-1}\rightarrow {\mathbb {R}}^\ell$ that sends exactly $b_i$ vertices of $S({\mathbf {1}}_{N})$ to $v_i$, $i=0,\ldots ,\ell$. Expression (5) implies that $\pi$ sends the origin to the origin, which in turn implies $\pi$ to be an integer projection. In particular,

$$\begin{aligned} \mu (P,{\mathbb {Z}}^\ell ) \le \mu _\ell \bigl (\pi (S({\mathbf {1}}_{N})),\pi ({\mathbb {Z}}^{N-1})\bigr ) \le \mu _\ell (S({\mathbf {1}}_{N}),{\mathbb {Z}}^{N-1}) = \frac{\ell }{2}, \end{aligned}$$

since $$\pi ({\mathbb {Z}}^{N-1}) \subseteq {\mathbb {Z}}^\ell $$. $$\square $$

### Conjecture A in Dimensions 2 and 3

We here prove Conjecture A in dimensions two and three, including the case of equality.$\square$

*Conjecture* *A**in dimension two*   Let $I=[-1,1]$ and $I'=[0,2]$ be intervals of length two centered at 0 and 1, respectively.

#### Lemma 3.3

The three polygons $S({\mathbf {1}}_3)$, $I \oplus I$, and $I \oplus I'$ have covering radius equal to 1.

#### Proof

For $S({\mathbf {1}}_3)$ this is just (1). For the other two polygons it follows from Corollary 2.2, since they are unimodularly equivalent to direct sums of segments of length two. $\square$

**Fig. 1:**
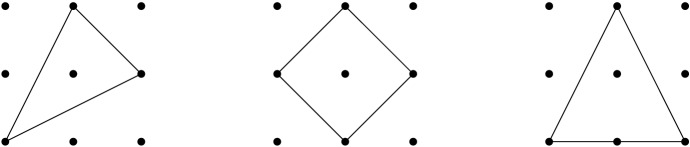
The non-hollow lattice polygons $S({\mathbf {1}}_3)$, $I \oplus I$, and $I\oplus I'$ of covering radius 1

We now show that every other non-hollow lattice polygon contains a (unimodularly equivalent) copy of one of these three, which implies Conjecture A. For this let us consider the following auxiliary family of lattice triangles with *k* interior lattice points: For each $k\in {\mathbb {N}}$, and $\alpha \in \{0,1\}$, let

$$\begin{aligned} M_k(\alpha )={{\,\mathrm{conv}\,}}{\{(-1,0), (1,\alpha ), (0, k+1)\}}. \end{aligned}$$

Observe that

$$\begin{aligned} M_1(0) = I\oplus I',\quad M_1(1) \cong S({\mathbf {1}}_3),\quad \text {and}\quad \forall \,k\ge 2, \ M_{k-1}(\alpha ) \subsetneq M_{k}(\alpha ). \end{aligned}$$

#### Remark 3.4

The covering radius of $M_k(\alpha )$ can be computed explicitly via

$$\begin{aligned} M_k(0) \cong I \oplus [0,k+1]\quad \text {and}\quad M_k(1) \cong S(k,1,1). \end{aligned}$$

Indeed, this implies

$$\begin{aligned} \mu (M_k(0))=\frac{1}{2}+\frac{1}{k+1}=\frac{k+3}{2k+2}\quad \text {and}\quad \mu (M_k(1)) = \frac{1+2/k}{2+1/k} = \frac{k+2}{2k+1}, \end{aligned}$$

by Corollary 2.2 and Theorem 1.4, respectively. We see that their covering radius equals 1 for $k=1$, and is strictly smaller for $k \ge 2$.

The following statement might be known in the literature on lattice polygons. In absence of a clear reference we give a detailed proof for the sake of a complete presentation.

#### Lemma 3.5

Every non-hollow lattice polygon *P* contains a unimodular copy of either $M_1(0)= I\oplus I'$, $M_1(1)\cong S({\mathbf {1}}_3)$, or $I\oplus I$.

#### Proof

Without loss of generality, assume the origin is in the interior of *P*. Consider the complete fan whose rays go through all non-zero lattice points in *P*. We call this the *lattice fan* associated to *P*, and it is a complete unimodular fan. Since a 2-dimensional fan is uniquely determined by its rays, we denote by ${\mathcal {F}}\{v_1,\dots ,v_m\}$ the fan with rays through $v_1,\dots ,v_m\in {\mathbb {R}}^2$. In particular, the lattice fan of *P* is denoted by ${\mathcal {F}}\{P\cap {\mathbb {Z}}^2\}$.

By the classification of complete unimodular fans, see [9, Thm. V.6.6], ${\mathcal {F}}\{P\cap {\mathbb {Z}}^2\}$ can be obtained (modulo unimodular equivalence) by successively refining the lattice fan of either $S({\mathbf {1}}_3)$ or

$$\begin{aligned} {\mathcal {F}}_l:= {\mathcal {F}}\{(0,-1), (0,1), (-1,0), (1,l)\}, \end{aligned}$$

for some $l\in {\mathbb {Z}}_{\ge 0}$. Observe that ${\mathcal {F}}_0$ is the lattice fan of $I\oplus I$, ${\mathcal {F}}_1$ refines the lattice fan of $S({\mathbf {1}}_3)\cong M_1(1)$ and, for every $l\ge 2,$ we have that ${\mathcal {F}}_l$ is unimodularly equivalent to the lattice fan of

$$\begin{aligned} {\left\{ \begin{array}{ll} M_k(0)&{} \text { if } l=2k \text { is even, and}\\ M_k(1)&{} \text { if } l=2k-1 \text { is odd,} \end{array}\right. } \end{aligned}$$

independently of which interior lattice point of $M_k(\alpha )$ we consider the rays of its lattice fan emanating from.

This, together with the fact that $M_1(\alpha ) \subseteq M_k(\alpha )$ for every $k\ge 1$, implies that *P* contains one of $M_1(0)$, $M_1(1)$, or $I\oplus I$. $\square$

#### Corollary 3.6

Let *P* be a non-hollow lattice polygon. Then

$$\begin{aligned} \mu (P)\le 1, \end{aligned}$$

with equality if and only if *P* is unimodularly equivalent to one of $S({\mathbf {1}}_3)$, $I \oplus I$, or $I \oplus I'$.

#### Proof

By Lemma 3.5, unless *P* is one of $S({\mathbf {1}}_3)$, $I \oplus I$, or $I \oplus I'$, it strictly contains one of them. If the latter happens then its covering radius is strictly smaller than 1, since the three of them are tight by Lemmas 2.7 and 2.8. $\square$

*Conjecture* *A**in dimension three*   For the three-dimensional case we introduce the following concept:

#### Definition 3.7

A *minimal* *d*-*polytope* is a non-hollow lattice *d*-polytope not properly containing any other non-hollow lattice *d*-polytope.

In this language, our results in dimension 2 can be restated as: There are exactly three minimal 2-polytopes, they have covering radius 1, and every other non-hollow lattice 2-polytope has strictly smaller covering radius.

In dimension three things are a bit more complicated. To start with, instead of three direct sums of (perhaps translated) simplices of the form $S({\mathbf {1}}_i)$ there are nine, that we now describe. As in the previous section, let $I=[-1,1]=S({\mathbf {1}}_2)$ and $I'=[0,2]$. In a similar way we define

$$\begin{aligned} S'({\mathbf {1}}_3)&= (1,1) + S({\mathbf {1}}_3) = {{\,\mathrm{conv}\,}}{\{(0,0),(2,1),(1,2)\}},\\ (I \oplus I')^\circ&= (0,-1) + (I \oplus I') = {{\,\mathrm{conv}\,}}{\{(0,1), (\pm 1,-1)\}},\\ (I \oplus I')'&= (0, -2) + (I \oplus I') = {{\,\mathrm{conv}\,}}{\{(0,0), (\pm 1,-2)\}}. \end{aligned}$$

Put differently, $S'({\mathbf {1}}_3)$ is $S({\mathbf {1}}_3)$ translated to have the origin as a vertex; the other two are $I \oplus I'$ translated to have the origin in the interior and at the “apex”, respectively.

#### Lemma 3.8

There are the following nine non-equivalent lattice 3-polytopes of covering radius 3/2, obtained as direct sums of (perhaps translated) simplices of the form $S({\mathbf {1}}_d)$:

$$\begin{aligned} \begin{array}{c} S({\mathbf {1}}_4),\\ S({\mathbf {1}}_3) \oplus I, \qquad S'({\mathbf {1}}_3) \oplus I, \qquad S({\mathbf {1}}_3) \oplus I',\\ I\oplus I\oplus I, \qquad I\oplus I\oplus I',\\ (I \oplus I')^\circ \oplus I, \qquad (I \oplus I')'\oplus I, \qquad (I \oplus I')^\circ \oplus I'. \end{array} \end{aligned}$$

**Fig. 2:**
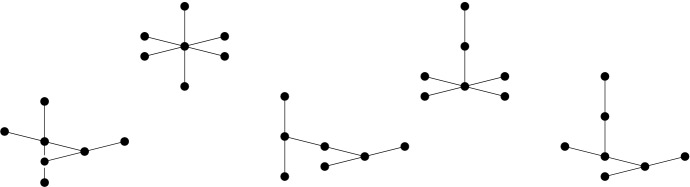
The five non-hollow lattice 3-polytopes that can be obtained by translations and direct sums of $I=[-1,1]$ arise as the convex hull of the shown segments

The last five polytopes are illustrated in Fig. 2, which is borrowed from [5, p. 123]. Observe that the last three can equally be written as

$$\begin{aligned} I \oplus (I \oplus I)', \qquad I \oplus (I' \oplus I)', \qquad I \oplus (I \oplus I')'', \end{aligned}$$

where $(I \oplus I)'$ denotes $I \oplus I$ translated to have the origin as a vertex and $(I \oplus I')''$ is $I \oplus I'$ translated to have the origin at an endpoint of its edge of length two.

#### Proof

That all the described direct sums are non-hollow follows from the following more general fact: The direct sum of two or more non-hollow lattice polytopes containing the origin is non-hollow if (and only if) all but at most one of the summands has the origin in its interior. Indeed, if the summand exists then its interior point(s) are interior in the sum; if it does not then the origin is an interior point in the sum. With this in mind, we only need to check that the nine described polytopes are pairwise unimodularly non-equivalent, which is left to the reader. $\square$

A second difference with dimension two is that these nine non-hollow lattice 3-polytopes are no longer the only minimal ones. Minimal non-hollow 3-polytopes have been classified and there are 26 with a single interior lattice point (see [18, Thm. 3.1] and Tables 2 & 4 therein) plus the infinite family described in Theorem 3.10.

To prove Conjecture A in dimension three we show that, on the one hand, the covering radii of the 26 with a single interior lattice point can be explicitly computed and/or bounded, giving the following result, the proof of which we postpone to Appendix C.

#### Theorem 3.9

Among the 26 minimal non-hollow 3-polytopes with a single interior lattice point, all except the nine in Lemma 3.83.8 have covering radius strictly smaller than 3/2.

On the other hand, all the (infinitely many) minimal non-hollow 3-polytopes with more than one interior lattice point have covering radius strictly smaller than 3/2, as we now prove. For any $k\in {\mathbb {N}}$ and $\alpha , \beta \in \{0,1\}$, we define $M_k(\alpha ,\beta )$ to be the following lattice tetrahedron:

$$\begin{aligned} M_k(\alpha ,\beta )= {{\,\mathrm{conv}\,}}{\{(1,0,0), (-1,0,\alpha ), (0,1,k+1), (0,-1, k+1-\beta )\}}. \end{aligned}$$

#### Theorem 3.10

([2, Prop. 4.2])  Every minimal 3-polytope with $k\ge 2$ interior lattice points is equivalent by unimodular equivalence or refinement of the lattice to $M_k(\alpha ,\beta )$ for some $\alpha ,\beta \in \{0,1\}$.

Theorem 3.10 is a version of [2, Prop. 4.2], although more explicit than the original one. An example where refinement is needed in the statement is $M_k(0,0)$ considered with respect to the lattice $\Lambda$ generated by ${\mathbb {Z}}^3$ and $(1/q, 1-1/q,0)$, with *q* and $k+1$ coprime. $M_k(0,0)$ is still minimal with respect to $\Lambda$ because it contains no point of $\Lambda \setminus {\mathbb {Z}}^3$.

#### Proof

Let *P* be a minimal lattice 3-polytope with more than one interior lattice point, and let *L* be a line containing two of them. Without loss of generality we assume that $L = \{(0,0,z) : z\in {\mathbb {R}}\}$ and $L\cap P$ is the segment between $(0,0,z_1)$ and $(0,0,z_2)$, with $z_1 \in [0,1)$ and $z_2\in (r,r+1]$ for some $r \in \{2,\ldots ,k\}$, so that *L* contains *r* interior lattice points of *P*.

**Claim 1** *The minimal faces of* *P* *containing respectively* $(0,0,z_1)$ *and* $(0,0,z_2)$ *are non-coplanar edges.* Let $F_1$ and $F_2$ be those faces. If one of them, say $F_1$, had dimension two, then ${{\,\mathrm{conv}\,}}{(F_1 \cup \{(0,0,r)\})}$ would be a non-hollow lattice polytope strictly contained in *P*. If one of them, say $F_1$, had dimension zero then necessarily $F_1 = \{(0,0,z_1)\}=\{(0,0,0)\}$. This would imply ${{\,\mathrm{conv}\,}}{(P\cap {\mathbb {Z}}^3 \setminus \{{\mathbf {0}}\})}$ to be a non-hollow lattice polytope strictly contained in *P*. Thus, $F_1$ and $F_2$ are both edges of *P*. They cannot be coplanar, since otherwise there would be vertices *p* and *q* of *P*, one on either side of the hyperplane ${{\,\mathrm{aff}\,}}{(F_1 \cup F_2)}$, and the polytope ${{\,\mathrm{conv}\,}}(F_1 \cup \{(0,0,r),p,q\})$ would be non-hollow and strictly contained in *P*. Hence, ${{\,\mathrm{conv}\,}}{(F_1 \cup F_2)}$ is a non-hollow lattice tetrahedron and, by minimality, $P={{\,\mathrm{conv}\,}}{(F_1 \cup F_2)}$. We denote as $v_i$ and $w_i$ the vertices of $F_i$, for $i=1,2$.

**Claim 2** *All the lattice points in the tetrahedron* *P* *other than the four vertices are on the line* *L*. Let $H_i$ be the plane containing the line *L* and the edge $F_i$, for $i=1,2$. The polytope $Q= {{\,\mathrm{conv}\,}}{(L\cap P\cup \{v_1,w_1,v_2\})}\subset P$ is contained in $H_1^+$, one of the two halfspaces defined by $H_1$; furthermore, the facet of *Q* lying on $H_1$ is non-hollow, since (0, 0, 1) is in its relative interior. Therefore, if *P* contained any lattice point *u* other than the vertex $w_2$ in the open halfspace $(H_1^-)^o$ then ${{\,\mathrm{conv}\,}}{(Q\cup \{u\})}$ would be a non-hollow lattice polytope strictly contained in *P*. Thus there are no lattice points in the open halfspace $(H_1^-)^o$. Since the same can be said for the other halfspaces, $H_1^+$ and $H_2^\pm$, all lattice points of *P* except its four vertices must lie on *L*. In particular, we have $r=k$.

**Claim 3** *The endpoint* $(0,0,z_i)$ *equals the mid-point of the edge* $F_i={{\,\mathrm{conv}\,}}{\{v_i,w_i\}}$. Let us only look at $i=1$, the other case being symmetric. Let $u_1=(0,0,1)$ and $u_2=(0,0,2)$ be the first two interior lattice points of *P* along *L*. The triangles ${{\,\mathrm{conv}\,}}{\{u_1,u_2,v_1\}}$ and ${{\,\mathrm{conv}\,}}{\{u_1,u_2,w_1\}}$ are empty lattice triangles in the plane $H_1$, hence they have the same area. Thus, $v_1$ and $w_1$ are at the same distance from (and on opposite sides of) the line *L*, which implies the statement. In particular, $z_1\in [0,1)$ and $z_2\in (k,k+1]$ are either integers or half-integers, so they can be written as $z_1=\alpha /2$ and $z_2=k+1-\beta /2$ for some $\alpha ,\beta \in \{0,1\}$. It is now clear that the affine transformation that fixes *L* and sends $v_1 \mapsto (1,0,0)$ and $v_2 \mapsto (0,1,k+1)$, sends *P* to $M_k(\alpha , \beta )$. The map may send ${\mathbb {Z}}^3$ to a different lattice $\Lambda$, but $\Lambda$ refines ${\mathbb {Z}}^3$ since (1, 0, 0), $(0,1,k+1)$, (0, 0, 1), and (0, 0, 2) are in $\Lambda$ and they generate ${\mathbb {Z}}^3$. $\square$

#### Corollary 3.11

Every minimal 3-polytope with $k\ge 2$ interior lattice points has covering radius strictly smaller than 3/2.

#### Proof

The projection of $M_k(\alpha ,\beta )$ along the *z* direction is $I\oplus I$ and the fiber over the origin is the segment $\{0\}\times \{0\} \times [\alpha /2, k+1 - \beta /2]$, of length $k+1 - (\alpha + \beta )/2$. Thus, by Lemma 2.1,

$$\begin{aligned} \mu (M_k(\alpha ,\beta ))\le \mu (I\oplus I)+\mu ([\alpha /2,k+1-\beta /2])=1+\frac{1}{k+1-(\alpha +\beta )/2}\le \frac{3}{2}. \end{aligned}$$

Moreover, the last inequality is met with equality only in the case $k=2$, $\alpha =\beta =1$. But for $M_2(1,1)$ we can consider the projection $(x,y,z) \mapsto x$, whose image is *I* and whose fiber is

$$\begin{aligned} {{\,\mathrm{conv}\,}}{\{(0,1/2), (1,3), (-1,2)\}}\cong S(3/2,1,1). \end{aligned}$$

Thus, by Lemma 2.1 and Theorem 1.4, we have

$$\begin{aligned} \qquad \qquad \mu (M_2(1,1)) \le \mu (I) + \mu (S(3/2,1,1)) = \frac{1}{2} + \frac{7/3}{8/3} = \frac{11}{8}<\frac{3}{2}. \,\,\qquad \square \end{aligned}$$

In fact, we can be more explicit:

#### Remark 3.12

The covering radius of $M_k(\alpha ,\beta )$ admits a closed expression:

$$\begin{aligned} \mu (M_k(0,0))&= \mu (I \oplus [0,k+1] \oplus I) = 1 + \frac{1}{k+1}.\\ \mu (M_k(1,0))&= \mu (M_k(0,1)) = \mu (I \oplus M_k(1))= 1 + \frac{3}{4k+2},\\ \mu (M_k(1,1))&= 1+ \frac{1}{2k}. \end{aligned}$$

The first formula directly follows from Lemma 2.1. The second one also does, using Remark 3.4. For the third one, see Lemma B.5 in Appendix B. For $k=1$ the three expressions reduce to 3/2, which follows also from $M_1(0,0) \cong I\oplus (I\oplus I')'$, $M_1(0,1) \cong I \oplus S({\mathbf {1}}_3)$, and $M_1(1,1) \cong S({\mathbf {1}}_4)$.

We are now ready to prove Conjecture A in dimension three:

#### Theorem 3.13

Let *P* be a non-hollow lattice 3-polytope. Then

$$\begin{aligned} \mu (P)\le \frac{3}{2}, \end{aligned}$$

with equality if and only if *P* is unimodularly equivalent to one of the nine polytopes in Lemma 3.83.8.

#### Proof

Let *P* be a non-hollow lattice 3-polytope, and let *T* be a minimal one contained in it. If *T* is not one of the nine in Lemma 3.83.8 then *T*, and hence *P*, has covering radius strictly smaller than 3/2 by either Corollary 3.11 or Theorem 3.9. If *T* is one of the nine and $P\ne T$ then

$$\begin{aligned} \mu (P)<\mu (T)=\frac{3}{2}, \end{aligned}$$

since these nine are tight by Lemmas 2.7 and 2.8. $\square$

## Conjecture C

We here focus on Conjecture C. We show that it implies Conjecture A, we prove it up to a factor of two in arbitrary dimension, and we prove it in dimension two. Finally, in Sect. 4.4, we investigate how the proposed bound changes if we allow the origin to be contained in the boundary of the given simplex.

As a preparation, let us first reinterpret Conjecture C in terms of (reciprocals of) certain lengths. To this end, let $S={{\,\mathrm{conv}\,}}{\{v_0,\dots ,v_d\}}$ be a *d*-simplex with the origin in its interior, and assume that it has *rational vertex directions*, that is, the line through the origin and the vertex $v_i$ has rational direction, for every $0\le i \le d$.

As in Conjecture C, let $\pi _i$ be the linear projection to dimension $d-1$ vanishing at $v_i$. Finally, let $\ell _i$ be the lattice length of $S\cap \pi _i^{-1}({\mathbf {0}})$. Put differently, let $u_i$ be the point where the ray from $v_i$ through ${\mathbf {0}}$ hits the opposite facet of *S* and let $\ell _i$ be the ratio between the length of $[u_i,v_i]$ and the length of the primitive lattice vector in the same direction. In formula:

$$\begin{aligned} \ell _i:={{\,\mathrm{Vol}\,}}_{{\mathbb {Z}}^d \cap {\mathbb {R}}v_i}[u_i, v_i]. \end{aligned}$$

### Lemma 4.1

For every $i \in \{0,1,\ldots ,d\}$, we have

$$\begin{aligned} \frac{1}{\ell _i} = \frac{ {{\,\mathrm{Vol}\,}}_{\pi _i({\mathbb {Z}}^d)}(\pi _i(S))}{{{\,\mathrm{Vol}\,}}_{{\mathbb {Z}}^d}S}. \end{aligned}$$

In particular, Conjecture C is equivalent to the inequality

6 $$\begin{aligned} \mu (S) \le \frac{1}{2} \sum _{i=0}^d \frac{1}{\ell _i}. \end{aligned}$$

### Proof

By construction, we have $\pi _i(S)=\pi _i(F_i)$, where $F_i$ is the facet of *S* opposite to the vertex $v_i$. Therefore, ${{\,\mathrm {vol}\,}}S={{\,\mathrm {vol}\,}}\pi _i(S){{\,\mathrm {vol}\,}}{[u_i,v_i]}/d$. The determinants of the involved lattices are related by $1=\det {\mathbb {Z}}^d=\det (\pi _i({\mathbb {Z}}^d))\det {({\mathbb {Z}}^d \cap {\mathbb {R}}v_i)}$ (cf. [22, Prop. 1.2.9]). Hence,

$$\begin{aligned} {{\,\mathrm{Vol}\,}}_{{\mathbb {Z}}^d}S&= \frac{d!{{\,\mathrm{vol}\,}}S}{\det {\mathbb {Z}}^d}= \frac{(d-1)! {{\,\mathrm{vol}\,}}(\pi _i(S))}{\det (\pi _i({\mathbb {Z}}^d))} \frac{{{\,\mathrm{vol}\,}}{[u_i,v_i]}}{\det {({\mathbb {Z}}^d\cap {\mathbb {R}}v_i)}} \\&= {{\,\mathrm{Vol}\,}}_{\pi _i({\mathbb {Z}}^d)}\left( \pi _i(S)\right) {{\,\mathrm{Vol}\,}}_{{\mathbb {Z}}^d \cap {\mathbb {R}}v_i}[u_i,v_i] \end{aligned}$$

as desired. $$\square $$

We now also detail the claim in the introduction, that the discrete surface area defined in Definition 1.5 is invariant under unimodular transformations.

### Lemma 4.2

Let *S* be a *d*-simplex with the origin in its interior and with rational vertex directions. Let *A* be an invertible linear transformation. Then

$$\begin{aligned} {{\,\mathrm{Surf}\,}}_{A{\mathbb {Z}}^d}\left( AS\right) = {{\,\mathrm{Surf}\,}}_{{\mathbb {Z}}^d}S. \end{aligned}$$

In particular, if *A* is unimodular, we have ${{\,\mathrm{Surf}\,}}_{{\mathbb {Z}}^d}(AS)={{\,\mathrm{Surf}\,}}_{{\mathbb {Z}}^d}S$.

### Proof

As before we write $S={{\,\mathrm{conv}\,}}{\{v_0,\ldots ,v_d\}}$ and we let $\pi _i$ be the projection vanishing at $v_i$, for $0\le i\le d$. Clearly, $AS={{\,\mathrm{conv}\,}}{\{Av_0,\ldots ,Av_d\}}$ and the corresponding projection ${\bar{\pi }}_i$ vanishing at $Av_i$ can be written as ${\bar{\pi }}_i=\pi _i A^{-1}$. Therefore, we get

$$\begin{aligned} {{\,\mathrm{Surf}\,}}_{A{\mathbb {Z}}^d}\left( AS \right) =\sum _{i=0}^d{{\,\mathrm{Vol}\,}}_{{\bar{\pi }}_i(A{\mathbb {Z}}^d)}{(\bar{\pi }}_i(AS))=\sum _{i=0}^d {{\,\mathrm{Vol}\,}}_{\pi _i(\mathbb Z^d)}\; (\pi _i(S))={{\,\mathrm{Surf}\,}}_{{\mathbb {Z}}^d}S \end{aligned}$$

as claimed. $$\square $$

### Conjecture C Implies Conjecture A

#### Corollary 4.3

Conjecture C$$\Rightarrow $$ Conjecture A.

#### Proof

In view of Corollary 3.2, it suffices to consider lattice simplices. Therefore, let $S={{\,\mathrm{conv}\,}}{\{v_0,\ldots ,v_d\}}$ be a lattice *d*-simplex containing the origin in its interior. Furthermore, let $\omega _i$ be the lattice length of the segment $[{\mathbf {0}},v_i]$. Then, $1- \omega _i/\ell _i$ is the *i*-th barycentric coordinate of the origin with respect to the vertices of *S*, so that

$$\begin{aligned} \sum _{i=0}^d \biggl (1- \frac{\omega _i}{\ell _i}\biggr ) = 1 \end{aligned}$$

and, hence, $\sum _{i=0}^d\omega _i/\ell _i=d$. On the other hand, for a lattice simplex we have $\omega _i\ge 1$. Thus, assuming Conjecture C holds for *S*, we have

$$\begin{aligned} \qquad \qquad \qquad \qquad \qquad \mu (S) \le \frac{1}{2} \sum _{i=0}^d \frac{1}{\ell _i}\le \frac{1}{2} \sum _{i=0}^d \frac{\omega _i}{\ell _i}=\frac{d}{2}. \quad \square \end{aligned}$$

### Conjecture C Holds up to a Factor of Two

In the formulation of Lemma 4.1, Conjecture C is easily proven inductively up to a factor of two.

#### Proposition 4.4

Let $S = {{\,\mathrm{conv}\,}}{\{v_0,\ldots ,v_d\}}$ be a *d*-simplex with the origin in its interior and with rational vertex directions. Then

$$\begin{aligned} \mu (S)\le \sum _{i=0}^d \frac{1}{\ell _i}, \end{aligned}$$

with the lattice lengths $\ell _i$ defined as above.

#### Proof

As above, let $u_i$ be the intersection of the line ${\mathbb {R}}v_i$ with the facet *F* of *S* opposite to $v_i$, so that $\ell _i$ is the lattice length of $Q:=[u_i,v_i]\subseteq S$. Note, that $u_i$ lies in the relative interior of *F*. Also, let $\pi _i$ be the linear projection vanishing at $v_i$. By the assumptions on *S*, the projection $\pi _i$ is rational and thus $\pi _i(S)$ is a $(d-1)$-dimensional simplex having the origin in its interior and with rational vertex directions with respect to $\pi _i({\mathbb {Z}}^d)$.

Using Lemma 2.1 and the induction hypothesis for $\pi _i(S)$, we get

7 $$\begin{aligned} \mu (S,{\mathbb {Z}}^d)&\le \mu (Q,{\mathbb {Z}}^d \cap L_Q)+\mu \bigl (\pi _i(S),\pi _i({\mathbb {Z}}^d)\bigr )\le \frac{1}{\ell _i}+\sum _{j\ne i}\frac{1}{\ell _j'}, \end{aligned}$$

where the $\ell _j'$ are the corresponding lattice-lengths in $\pi _i(S)$. Thus, to prove the proposition we only need to show that $\ell _j' \ge \ell _j$, for all $j \ne i$. In fact, since the one-dimensional lattice $\pi _i({\mathbb {Z}}^d)\cap \pi _i({\mathbb {R}}v_j)$ refines $\pi _i({\mathbb {Z}}^d \cap {\mathbb {R}}v_j)$, we have

$$\begin{aligned} \ell _j&= {{\,\mathrm{Vol}\,}}_{{\mathbb {Z}}^d \cap {\mathbb {R}}v_j}[u_j, v_j]={{\,\mathrm{Vol}\,}}_{\pi _i({\mathbb {Z}}^d \cap {\mathbb {R}}v_j)}[\pi _i(u_j), \pi _i(v_j)]\\&\le {{\,\mathrm{Vol}\,}}_{\pi _i({\mathbb {Z}}^d) \cap \pi _i({\mathbb {R}}v_j)}[\pi _i(u_j), \pi _i(v_j)]\le \ell _j'. \end{aligned}$$

Here, the last inequality comes from the fact that $[\pi _i(u_j), \pi _i(v_j)] \subseteq \pi _i(S)$ is contained in the ray from the vertex $\pi _i(v_j)$ of $\pi _i(S)$ through the origin. $\square$

#### Remark 4.5

Corollary 4.11 in the next section proves Conjecture C in the plane. So we can base the inductive proof above on the stronger assumption that $\mu (S') \le c_{d-1} \sum _{i=0}^{d-1}1/\ell _i'$, where $S'$ is a $(d-1)$-dimensional simplex and $c_{d-1}$ is a suitable constant with $c_2=1/2$. Summing the thus modified inequality (7) for all indices $0 \le i \le d$, yields the recursion $(d+1) c_d = 1+d c_{d-1}$. Solving it shows that

$$\begin{aligned} \mu (S)\le \frac{2d-1}{2d+2}\sum _{i=0}^d\frac{1}{\ell _i} \end{aligned}$$

for all *d*-simplices *S* with the origin in its interior and with rational vertex directions. This is a good bound in ${\mathbb {R}}^3$ since $c_3 = 5/8$.

### Conjecture C in Dimension Two

In this section we prove Conjecture C in dimension two. Our first remarks are valid in arbitrary dimension.

Throughout, let $S = {{\,\mathrm{conv}\,}}{\{v_0,\ldots ,v_d\}}$ be a simplex with the origin in its interior and with rational vertex directions. For each $i=0,\ldots ,d$, let $p_i$ be the primitive positive multiple of $v_i$. Let $\alpha = (\alpha _0,\dots ,\alpha _d)\in {\mathbb {N}}^{d+1}$ be the primitive integer linear dependence among the $p_i$’s. That is,

$$\begin{aligned} \sum _{i=0}^d \alpha _i p_i = {\mathbf {0}}\qquad \text {and} \qquad \gcd {(\alpha _0,\dots ,\alpha _d)} = 1. \end{aligned}$$

Denoting the Euclidean length of a vector $x\in {\mathbb {R}}^d$ by $\Vert x\Vert$, and writing $\beta _i=\alpha _i\Vert p_i\Vert /\Vert v_i\Vert \in {\mathbb {R}}_{>0}$, for each $i=0,\ldots ,d$, we have

$$\begin{aligned} \sum _{i=0}^d \beta _i v_i = \sum _{i=0}^d \alpha _i p_i = {\mathbf {0}}. \end{aligned}$$

#### Remark 4.6

The fact that the $p_i$’s are primitive imposes some condition on the vector $\alpha \in {\mathbb {N}}^{d+1}$. Namely, for each $i\in \{0,\ldots ,d\}$, we have

$$\begin{aligned} \gcd {(\alpha _j : j\ne i)} = 1. \end{aligned}$$

Indeed, let $\Lambda$ be the lattice generated by $\{p_0,p_1,\ldots ,p_d\}$, and let $\Lambda _i$ be the sublattice generated by $\{p_j : j \ne i\}$. Then, the primitive vector of $\Lambda _i$ in the direction of $p_i$ is

$$\begin{aligned} \frac{\sum _{j\ne i}\alpha _j p_j}{\gcd {(\alpha _j : j\ne i)}}=\frac{- \alpha _i p_i}{\gcd {(\alpha _j : j\ne i)}}, \end{aligned}$$

which is an integer multiple of $p_i$ if, and only if, $\gcd (\alpha _j : j\ne i)= 1$.

As in the previous sections, for each *i* let $\ell _i$ be the lattice length of $S\cap {\mathbb {R}}v_i$. The following lemma says that the vectors $\alpha$ and $\beta =(\beta _0,\beta _1,\ldots ,\beta _d)$ contain all the information about *S* needed to compute the right-hand side in (6).

#### Lemma 4.7

The lattice length of $S \cap {\mathbb {R}}v_i$ equals

$$\begin{aligned} \ell _i=\frac{\alpha _i}{\beta _i}+\frac{\alpha _i}{\sum _{j\ne i}\beta _j}=\frac{\alpha _i}{\beta _i}\cdot \frac{\sum _{j=0}^d\beta _j}{\sum _{j\ne i}\beta _j}. \end{aligned}$$

#### Proof

To slightly simplify notation, we do the computations for $i=0$. For this, let us use the vectors $p_1,\dots ,p_d$ as the basis for a linear coordinate system in ${\mathbb {R}}^d$. In these coordinates, $p_0$ becomes

$$\begin{aligned} p_0 = - \frac{1}{\alpha _0}( \alpha _1,\dots ,\alpha _d). \end{aligned}$$

On the other hand, the equation of the facet of *S* opposite to $v_0$ is

$$\begin{aligned} \sum _{j=1}^d \frac{\beta _j}{\alpha _j} x_j = 1, \end{aligned}$$

so that this facet intersects the line spanned by $p_0$ at the point

8 $$\begin{aligned} \frac{(\alpha _1,\dots ,\alpha _d)}{\sum _{j=1}^d \beta _j}=\frac{-\alpha _0}{\sum _{j=1}^d\beta _j} p_0. \end{aligned}$$

Thus, the segment $S \cap {\mathbb {R}}v_0$ has endpoints $({\alpha _0}/{\beta _0}) p_0$ and (8), which implies the statement. $\square$

#### Remark 4.8

Observe that the quantity $\omega _i$ in the proof of Corollary 4.3 equals $\alpha _i/\beta _i$. With this in mind, one easily recovers the equality $\sum _i{\omega _i/\ell _i}=d$ used in that proof, from Lemma 4.7.

*Specializing to dimension two*   Our proof of Conjecture C in two dimensions is based on applying Lemma 2.1 to the projection $\pi :{\mathbb {R}}^2\rightarrow {\mathbb {R}}$ along the direction of $v_i$, for some fixed $i\in \{0,1,2\}$. Then, with the notation above,

(i) $\alpha _0$, $\alpha _1$, and $\alpha _2$ are pairwise coprime, by Remark 4.6;
(ii) the lattice length of $S\cap \pi ^{-1}({\mathbf {0}})$ is $\ell _i$;
(iii) the lattice length of $\pi (S)$ equals
  $$\begin{aligned} \frac{\alpha _j\alpha _k}{\beta _j}+ \frac{\alpha _j\alpha _k}{\beta _k} =\frac{\alpha _j\alpha _k}{\beta _j\beta _k}(\beta _j + \beta _k), \end{aligned}$$
  where $\{j,k\}=\{0,1,2\}\setminus \{i\}$. Here we use that the projection of the segment $[{\mathbf {0}},v_j]=(\alpha _j/\beta _j)[{\mathbf {0}},p_j]$ has length $\alpha _k\alpha _j/\beta _j$, since $\gcd (\alpha _j,\alpha _k)=1$ implies that $\pi (p_j/\alpha _k)$ is a primitive lattice point in the projection.

Writing $L=\pi ^{-1}({\mathbf {0}})$, Lemma 2.1 gives us

$$\begin{aligned} \mu (S) \le \mu (S\cap L,{\mathbb {Z}}^2 \cap L) + \mu \bigl (\pi (S),\pi ({\mathbb {Z}}^2)\bigr ). \end{aligned}$$

Hence, the inequality (6) would follow from

9 $$\begin{aligned} \frac{1}{\ell _j} + \frac{1}{\ell _k} - \frac{1}{\ell _i} \ge \frac{2\beta _j\beta _k}{\alpha _j\alpha _k(\beta _j + \beta _k)}. \end{aligned}$$

We prove this inequality under mild assumptions.

#### Theorem 4.9

Let $S={{\,\mathrm{conv}\,}}{\{v_0,v_1,v_2\}}\subseteq {\mathbb {R}}^2$ be a triangle with the origin in its interior and with rational vertex directions. Let the vectors $\alpha$ and $\beta$, and the lengths $\ell _i$ be defined as above, and let $p_0$, $p_1$, and $p_2$ be primitive in the directions of $v_0$, $v_1$, and $v_2$. Assume that $(\alpha _0,\alpha _1,\alpha _2)\ne (1,1,1)$. Then, the inequality (9) holds for some choice of $i\in \{0,1,2\}$. Moreover, the inequality is strict unless $(\alpha _0,\alpha _1,\alpha _2) = (2,1,1)$ and $\beta _1=\beta _2$, up to reordering the indices.

#### Example 4.10

(i) The necessity of $(\alpha _0,\alpha _1,\alpha _2) \ne (1,1,1)$ is shown by the following example. If $S=S(1,1,1)$ (so that $\alpha _i=\beta _i=1$ for all *i*), then
  $$\begin{aligned} \frac{1}{\ell _j}+\frac{1}{\ell _k}-\frac{1}{\ell _i}=\frac{2}{3}\qquad \text {and} \qquad \frac{2\beta _j \beta _k}{\alpha _j \alpha _k(\beta _k + \beta _k)}=1, \end{aligned}$$
  so the inequality fails.
(ii) Even if $(\alpha _0,\alpha _1,\alpha _2) \ne (1,1,1)$, it is not true that (9) holds *for every* $i\in \{0,1,2\}$. For $\omega >0$, consider the simplex
  $$\begin{aligned} S = {{\,\mathrm{conv}\,}}{\{(0,\omega ), (-1,-1), (1,-1)\}}. \end{aligned}$$
  It has parameters $(\alpha _0, \alpha _1, \alpha _2) = (2,1,1)$, $(\beta _0, \beta _1, \beta _2) = (2/{\omega }, 1,1)$, $\ell _0=\omega +1$, and $\ell _1=\ell _2=(2\omega +2)/(\omega +2)$. For $i=0$, we indeed have
  $$\begin{aligned} \frac{1}{\ell _1} + \frac{1}{\ell _2} - \frac{1}{\ell _0} = 1=\frac{2\beta _1\beta _2}{\alpha _1\alpha _2(\beta _1 + \beta _2)}. \end{aligned}$$
  But for $i \in \{1,2\}$, we get
  $$\begin{aligned} \frac{1}{\ell _j} + \frac{1}{\ell _k} - \frac{1}{\ell _i} = \frac{1}{\ell _0} = \frac{1}{\omega +1}<\frac{2}{\omega +2}=\frac{2\beta _j\beta _k}{\alpha _j\alpha _k(\beta _j + \beta _k)}. \end{aligned}$$

#### Proof of Theorem 4.9

*Case 1: At most one of the* $\alpha _i$*s equals* 1. Say $\alpha _1\ne 1\ne \alpha _2$. With no loss of generality assume $\ell _2\ge \ell _1$. Then, by Lemma 4.7,

$$\begin{aligned} \frac{1}{\ell _0} + \frac{1}{\ell _1} - \frac{1}{\ell _2}\ge \frac{1}{\ell _0}= \frac{\beta _0}{\alpha _0} \cdot \frac{\beta _1 +\beta _2}{\beta _0+\beta _1+\beta _2} > \frac{\beta _0}{\alpha _0} \cdot \frac{\beta _1}{\beta _0 + \beta _1}\ge \frac{2\beta _0\beta _1}{\alpha _0\alpha _1(\beta _0 + \beta _1)}. \end{aligned}$$

*Case 2: Two of the* $\alpha _i$*s equal* 1. Assume that $\alpha _1=\alpha _2=1$. The condition $(\alpha _0,\alpha _1,\alpha _2)\ne (1,1,1)$ then implies $\alpha _0\ge 2$, so that Lemma 4.7 gives

$$\begin{aligned} \frac{1}{\ell _1} + \frac{1}{\ell _2} - \frac{1}{\ell _0}&= \frac{\beta _1(\beta _0 + \beta _2)}{\beta _0+\beta _1+\beta _2}+ \frac{\beta _2(\beta _0 + \beta _1)}{\beta _0+\beta _1+\beta _2}- \frac{\beta _0}{ \alpha _0} \cdot \frac{\beta _1 + \beta _2}{\beta _0+\beta _1+\beta _2} \\&= \frac{2 \beta _1\beta _2+(1-1/{\alpha _0})\beta _0 (\beta _1 +\beta _2)}{\beta _0+\beta _1+\beta _2}\overset{*}{\ge }\frac{2 \beta _1\beta _2+ \beta _0 (\beta _1 +\beta _2)/2}{\beta _0+\beta _1+\beta _2}. \end{aligned}$$

Thus, the inequality we want to prove is

$$\begin{aligned} \frac{2 \beta _1\beta _2+\beta _0 (\beta _1 +\beta _2)/2}{\beta _0+\beta _1+\beta _2} \ge \frac{2\beta _1\beta _2}{\beta _1 + \beta _2} \end{aligned}$$

or, equivalently,

$$\begin{aligned} 2 \beta _1\beta _2 (\beta _1 +\beta _2)+ \frac{\beta _0 (\beta _1 +\beta _2)^2}{2} \ge 2\beta _1\beta _2 (\beta _0+\beta _1+\beta _2). \end{aligned}$$

This is equivalent to $(\beta _1 +\beta _2)^2 \overset{*}{\ge } 4 \beta _1\beta _2$, which clearly holds.

The two inequalities we used, marked with “$\overset{*}{\ge }$”, are equalities if and only if $\alpha _0 =2$ and $\beta _1=\beta _2$, respectively. $\square$

We now prove Conjecture C for $d=2$ which also gives another proof of Conjecture A in the plane.

#### Corollary 4.11

Conjecture C holds in dimension two.

#### Proof

Let $S={{\,\mathrm{conv}\,}}{\{v_0,v_1,v_2\}}\subseteq {\mathbb {R}}^2$ be a triangle with the origin in its interior and with rational vertex directions. Let the vectors $\alpha$ and $\beta$, and the lengths $\ell _i$ be defined as above, taking $p_0$, $p_1$, and $p_2$ primitive. In view of Lemma 4.1 we need to show that

$$\begin{aligned} \mu (S) \le \frac{1}{2}\biggl ( \frac{1}{\ell _0} + \frac{1}{\ell _1} + \frac{1}{\ell _2} \biggr ). \end{aligned}$$

If $(\alpha _0,\alpha _1,\alpha _2)=(1,1,1)$, then consider the lattice $\Lambda$ generated by $p_0,p_1,p_2$. Let *A* be the linear transformation sending $e_i$ to $p_i$, for $i=1,2$. Then, $\Lambda =A{\mathbb {Z}}^2$ and $S=AS(\omega )$ for a suitable $\omega \in {\mathbb {R}}^3_{>0}$. Moreover, since the $p_i$s are primitive, the lattice lengths $\ell _i$ are the same for every pair $(S,{\mathbb {Z}}^2)$, $(S,\Lambda )$, and $(S(\omega ),{\mathbb {Z}}^2)$. Observing that $\Lambda \subseteq {\mathbb {Z}}^2$ is a sublattice, we may therefore apply Theorem 1.4 and get

$$\begin{aligned} \mu (S) \le \mu (S,\Lambda )=\mu (S(\omega ),{\mathbb {Z}}^2)=\frac{1}{2}\biggl ( \frac{1}{\ell _0} + \frac{1}{\ell _1} + \frac{1}{\ell _2} \biggr ). \end{aligned}$$

So, we assume that $(\alpha _0,\alpha _1,\alpha _2)\ne (1,1,1)$ and thus we can apply Theorem 4.9, which provides us with an index $i\in \{0,1,2\}$ such that the inequality (9) holds. As we saw above, this implies the desired bound. $\square$

### Analogs to Conjecture C with the Origin in the Boundary

As we said in the introduction, the question analogous to Conjecture A for general lattice polytopes has an easy answer: the maximum covering radius among all *d*-dimensional lattice polytopes equals *d* and is attained by, and only by, unimodular simplices. This phenomenon generalizes to analogs of Theorem 1.4 and Conjecture C, which admit easy proofs. The generalization concerns the simplices $S(\omega )$, except we now allow one of the entries of $\omega$ (typically the first one) to be zero so that the origin becomes a vertex:

#### Proposition 4.12

For an $\omega \in {\mathbb {R}}^{d}_{>0}$ let

$$\begin{aligned} S(0,\omega ):={{\,\mathrm{conv}\,}}{\{{\mathbf {0}}, \omega _1 e_1, \ldots , \omega _d e_d\}}. \end{aligned}$$

Then

$$\begin{aligned} \mu (S(0,\omega ))=\sum _{i=1}^d \frac{1}{\omega _i}=\frac{\sum _{i=1}^d {{\,\mathrm{Vol}\,}}_{\pi _i({\mathbb {Z}}^d)}\left( \pi _i(S(\omega ))\right) }{{{\,\mathrm{Vol}\,}}_{{\mathbb {Z}}^d}(S(\omega ))}, \end{aligned}$$

where $\pi _i:{\mathbb {R}}^d \rightarrow {\mathbb {R}}^{d-1}$ is the linear projection that forgets the *i*-th coordinate.

#### Proof

$S(0,\omega )$ can be redescribed as

$$\begin{aligned} \left\{ x\in {\mathbb {R}}_{\ge 0}^d : \sum _{i=1}^d \frac{x_i}{\omega _i} \le 1\right\} . \end{aligned}$$

In this form it is clear that $\mu (S(0,\omega ))$ equals the unique $\mu \in [0,\infty )$ such that ${\mathbf {1}}_d$ lies in the boundary of $\mu \cdot S(0,\omega )$, which equals $\sum _i1/{\omega _i}$, as stated. $\square$

#### Corollary 4.13

Let $S={{\,\mathrm{conv}\,}}{\{{\mathbf {0}},v_1,\ldots ,v_d\}}\subseteq {\mathbb {R}}^d$ be a *d*-simplex with rational vertex directions. For each $i=1,\ldots ,d$, let $\pi _i:{\mathbb {R}}^d \rightarrow {\mathbb {R}}^{d-1}$ be the linear projection vanishing at $v_i$. Then

$$\begin{aligned} \mu (S) \le \frac{\sum _{i=1}^d {{\,\mathrm{Vol}\,}}_{\pi _i({\mathbb {Z}}^d)}\left( \pi _i(S)\right) }{{{\,\mathrm{Vol}\,}}_{{\mathbb {Z}}^d}S}, \end{aligned}$$

with equality if and only if *S* is unimodularly equivalent (by a transformation fixing the origin) to $S(0,\omega )$ for some $\omega \in {\mathbb {R}}_{> 0}^d$.

#### Proof

Let $p_1,\ldots ,p_d \in {\mathbb {Z}}^d$ be the primitive vertex directions of *S*, so that $v_i=\omega _ip_i$, where $\omega _i$ is the lattice length of the segment $[{\mathbf {0}},v_i]$, for each $i=1,\ldots ,d$. Then, the linear map sending $p_i\mapsto e_i$, $i=1,\ldots ,d$, sends *S* to $S(0,\omega )$ and ${\mathbb {Z}}^d$ to a lattice $\Lambda$ containing ${\mathbb {Z}}^d$. This implies

$$\begin{aligned} \mu (S, {\mathbb {Z}}^d)= \mu (S(0,\omega ), \Lambda ) \le \mu (S(0,\omega ),{\mathbb {Z}}^d) = \frac{\sum _{i=1}^d {{\,\mathrm{Vol}\,}}_{\pi _i({\mathbb {Z}}^d)}\left( \pi _i(S)\right) }{{{\,\mathrm{Vol}\,}}_{{\mathbb {Z}}^d}S}, \end{aligned}$$

by Proposition 4.12.

The ‘if’ in the equality case is obvious: in this case $\Lambda = {\mathbb {Z}}^d$. For the ‘only if’ suppose that $\Lambda$ is a proper superlattice of ${\mathbb {Z}}^d$ and let $p\in \Lambda \cap [0,1)^d\setminus \{{\mathbf {0}}\}$ be a non-zero lattice point in the half-open unit cube. Let

$$\begin{aligned} \mu =\mu (S(0,\omega ),{\mathbb {Z}}^d)=\frac{\sum _{i=1}^d{{\,\mathrm{Vol}\,}}_{\pi _i({\mathbb {Z}}^d)}\left( \pi _i(S)\right) }{{{\,\mathrm{Vol}\,}}_{{\mathbb {Z}}^d}S}. \end{aligned}$$

Then the point ${\mathbf {1}}$ is the only point in the unit cube $[0,1]^d$ that is last covered by ${\mathbb {Z}}^d + \mu \cdot S(0,\omega )$. Since ${\mathbf {1}}$ lies in the interior of $p + \mu \cdot S(0,\omega )$, the covering radius of $S(0,\omega )$ is strictly smaller with respect to $\Lambda$ than it is with respect to ${\mathbb {Z}}^d$. $\square$

Our next results say that Proposition 4.12 and Corollary 4.13 are not only analogs (without the factor of two) of Theorem 1.4 and Conjecture C, but also a limit of them when we make one of the vertices tend to zero. We consider this as additional evidence for Conjecture C. Formally:

#### Theorem 4.14

Let $S= {{\,\mathrm{conv}\,}}{\{v_0,\ldots ,v_d\}}$ be a *d*-simplex with the origin in its interior and with rational vertex directions. For each $i\in \{0,\ldots ,d\}$ consider the one-parameter family of simplices

$$\begin{aligned} S^{(i)}_t:= {{\,\mathrm{conv}\,}}{\{v_0,\ldots ,tv_i,\ldots ,v_d\}}, \quad \ \ t \in [0,1], \end{aligned}$$

so that $S^{(i)}_1=S$ and $S^{(i)}_0={{\,\mathrm{conv}\,}}{\{v_1,\ldots ,{\mathbf {0}},\ldots ,v_d\}}$. For each $i=0,\dots ,d$ let $\pi _i:{\mathbb {R}}^d\rightarrow {\mathbb {R}}^{d-1}$ be the linear projection vanishing at $v_i$. Then, there is an index $j \in \{0,\ldots ,d\}$ such that

10 $$\begin{aligned} \lim _{t\rightarrow 0} \,\frac{1}{2}\cdot \frac{\sum _{i=0}^d {{\,\mathrm{Vol}\,}}_{\pi _i({\mathbb {Z}}^d)}(\pi _i(S^{(j)}_t))}{{{\,\mathrm{Vol}\,}}_{{\mathbb {Z}}^d}S^{(j)}_t}\ge \frac{\sum _{i=0, i\ne j}^d {{\,\mathrm{Vol}\,}}_{\pi _i({\mathbb {Z}}^d)}(\pi _i(S^{(j)}_0))}{{{\,\mathrm{Vol}\,}}_{{\mathbb {Z}}^d}S^{(j)}_0}, \end{aligned}$$

with equality if and only if the primitive lattice vectors parallel to $v_0,\ldots ,v_d$ add up to zero.

Observe that the condition for equality includes, but is more general than, the case when *S* is of the form $S(\omega )$.

#### Proof

For each *i*, let $u_i$ be the primitive lattice vector parallel to $v_i$, and let $U=\{u_0,\ldots ,u_d\}$. We choose *j* to be an index minimizing the (absolute value of the) determinant of $U \setminus \{u_i\}$ among all *i*. Observe that *S* is of the form $S(\omega )$ if and only if all those determinants are equal to 1.

To simplify notation, in the rest of the proof we assume $j=0$ and we drop the superindex from the notation $S^{(j)}_t$. Since the volume functional is continuous, we have

$$\begin{aligned} \lim _{t\rightarrow 0} {{{\,\mathrm{Vol}\,}}_{{\mathbb {Z}}^d}S_t} = {{{\,\mathrm{Vol}\,}}_{{\mathbb {Z}}^d}S_0}, \end{aligned}$$

and, for each $i=1,\dots ,d$,

$$\begin{aligned} \lim _{t\rightarrow 0} {{\,\mathrm{Vol}\,}}_{\pi _i({\mathbb {Z}}^d)}\!(\pi _i(S_t)) = {{\,\mathrm{Vol}\,}}_{\pi _i({\mathbb {Z}}^d)}\!(\pi _i(S_0)). \end{aligned}$$

Thus, the only thing to prove is that

$$\begin{aligned} \lim _{t\rightarrow 0} {{\,\mathrm{Vol}\,}}_{\pi _0({\mathbb {Z}}^d)}\!(\pi _0(S_t))\!\ge \sum _{i=1}^d {{\,\mathrm{Vol}\,}}_{\pi _i({\mathbb {Z}}^d)}(\pi _i(S_0)). \end{aligned}$$

The volume on the left-hand side does not depend on *t* because the vertex of $S_t$ that depends on *t* is projected out by $\pi _0$. Moreover, this volume equals $\sum _{i=1}^d{{\,\mathrm{Vol}\,}}_{(\pi _0({\mathbb {Z}}^d))}(\pi _0(F_i))$, where $F_i$ is the facet of $S_0$ opposite to $v_i$. Similarly, ${{\,\mathrm{Vol}\,}}_{\pi _i({\mathbb {Z}}^d)}(\pi _i(S_0))= {{\,\mathrm{Vol}\,}}_{\pi _i({\mathbb {Z}}^d)}(\pi _i(F_i))$. Hence, the inequality follows from

11 $$\begin{aligned} {{\,\mathrm{Vol}\,}}_{\pi _0({\mathbb {Z}}^d)}(\pi _0(F_i))\ge {{\,\mathrm{Vol}\,}}_{\pi _i({\mathbb {Z}}^d)}(\pi _i(F_i)). \end{aligned}$$

Both sides of (11) are integer multiples of ${{\,\mathrm{Vol}\,}}_{{\mathbb {Z}}^d\cap {{\,\mathrm{aff}\,}}F_i}F_i$, with the proportionality factors being the lattice distances from $F_i$ to $u_0$ and to $u_i$, respectively. These distances are proportional to the determinants of $U\setminus \{u_i\}$ and $U\setminus \{u_0\}$, so our assumption on $u_0$ minimizing this implies the statement. Moreover, we have equality if, and only if, all the determinants of $U\setminus \{u_i\}$ are equal to that of $U\setminus \{u_0\}$. This in turn is equivalent to $\sum _{i=0}^d u_i={\mathbf {0}}$. $\square$

#### Corollary 4.15

In the conditions of Theorem 4.14 and for the index *j* mentioned therein, we have

$$\begin{aligned} \lim _{t\rightarrow 0} \mu (S^{(j)}_t) \le \lim _{t\rightarrow 0}\,\frac{1}{2}\cdot \frac{\sum _{i=0}^d {{\,\mathrm{Vol}\,}}_{\pi _i({\mathbb {Z}}^d)}(\pi _i(S^{(j)}_t))}{{{\,\mathrm{Vol}\,}}_{{\mathbb {Z}}^d}S^{(j)}_t}, \end{aligned}$$

with equality if and only if the primitive lattice vectors parallel to $v_0,\dots ,v_d$ add up to zero.

#### Proof

This follows from Theorem 4.14 since

$$\begin{aligned} \lim _{t\rightarrow 0} \mu (S^{(j)}_t)=\mu (S^{(j)}_0)\le \frac{\sum _{i=0,i \ne j}^d {{\,\mathrm{Vol}\,}}_{\pi _i({\mathbb {Z}}^d)}(\pi _i(S^{(j)}_0))}{{{\,\mathrm{Vol}\,}}_{{\mathbb {Z}}^d}S^{(j)}_0}, \end{aligned}$$

where the last inequality is Corollary 4.13. $\square$

#### Remark 4.16

Equation (10) is not true for all choices of *j*. Without any assumption on *j* the proof of Theorem 4.14 carries through up to the point where we say that (10) would follow from (11), but the latter inequality is not true in general. For a specific example, let $S={{\,\mathrm{conv}\,}}{\{(0,-1), (1,1), (-1,1)\}}$ and consider $j=0$. Then for $i=1,2$,

$$\begin{aligned} {{\,\mathrm{Vol}\,}}_{\pi _0({\mathbb {Z}}^d)}(\pi _0(F_i)) = 1 < 2 = {{\,\mathrm{Vol}\,}}_{\pi _i({\mathbb {Z}}^d)}(\pi _i(F_i)). \end{aligned}$$

This gives

$$\begin{aligned} \lim _{t\rightarrow 0}\,\frac{1}{2}\cdot \frac{\sum _{i=0}^d{{\,\mathrm{Vol}\,}}_{\pi _i({\mathbb {Z}}^d)}(\pi _i(S^{(0)}_t))}{{{\,\mathrm{Vol}\,}}_{{\mathbb {Z}}^d}S^{(0))}_t}=\frac{1}{2}\cdot \frac{2+2+2}{2}=\frac{3}{2}, \end{aligned}$$

and

$$\begin{aligned} \frac{\sum _{i=1}^d {{\,\mathrm{Vol}\,}}_{\pi _i({\mathbb {Z}}^d)}(\pi _i(S^{(0)}_0))}{{{\,\mathrm{Vol}\,}}_{{\mathbb {Z}}^d}S^{(0)}_0} = \frac{2+2}{2} = 2. \end{aligned}$$

We finally look at the intermediate case where ${\mathbf {0}}$ is in the boundary of $S={{\,\mathrm{conv}\,}}{\{v_0,\ldots ,v_d\}}$ but not a vertex. We can generalize Conjecture C to

#### Conjecture E

Let $S = {{\,\mathrm{conv}\,}}{\{v_0,\ldots ,v_d\}}$ be a *d*-simplex with ${\mathbf {0}}\in S\setminus \{v_0,\ldots ,v_d\}$, and with rational vertex directions. Let $\pi _i:{\mathbb {R}}^d\rightarrow {\mathbb {R}}^{d-1}$ be the linear projection vanishing at $v_i$. Let $I\subset \{0,\dots ,d\}$ be the set of labels of facets of *S* containing ${\mathbf {0}}$. Then

12 $$\begin{aligned} \mu (S) \le \frac{1}{2}\cdot \frac{\sum _{i=0}^d{{\,\mathrm{Vol}\,}}_{\pi _i({\mathbb {Z}}^d)}(\pi _i(S)) + \sum _{i\in I} {{\,\mathrm{Vol}\,}}_{\pi _i({\mathbb {Z}}^d)}\left( \pi _i(S)\right) }{{{\,\mathrm{Vol}\,}}_{{\mathbb {Z}}^d}S}. \end{aligned}$$

#### Proposition 4.17

Conjecture E$$\Leftrightarrow $$ Conjecture C.

#### Proof

The implication Conjecture E$\Rightarrow$ Conjecture C is obvious, since the latter is the case $I=\emptyset$ of the former. For the other implication, for each $i=0,\ldots ,d$, let

$$\begin{aligned} \ell _i =\frac{{{\,\mathrm{Vol}\,}}_{{\mathbb {Z}}^d}S}{{{\,\mathrm{Vol}\,}}_{\pi _i({\mathbb {Z}}^d)}\left( \pi _i(S)\right) }, \end{aligned}$$

which equals the lattice length of the segment $S \cap {{\,\mathrm{lin}\,}}{\{v_i\}}$. The inequality in Conjecture E we want to prove becomes

$$\begin{aligned} \mu (S) \le \frac{1}{2} \sum _{i\notin I} \frac{1}{\ell _i} + \sum _{i\in I} \frac{1}{\ell _i}. \end{aligned}$$

Let $S_I= {{\,\mathrm{conv}\,}}{\{v_i : i \notin I\}}$ and $S_{{\overline{I}}}={{\,\mathrm{conv}\,}}{(\{{\mathbf {0}}\} \cup \{v_i: i \in I\})}$. Observe that $S_I$ equals the intersection of the facets of *S* containing ${\mathbf {0}}$, hence it is a $(d-|I|)$-simplex with ${\mathbf {0}}$ in its relative interior. $S_{{\overline{I}}}$ is an |*I*|-simplex with ${\mathbf {0}}$ as a vertex. Hence, Conjecture C and Proposition 4.12 respectively say:

$$\begin{aligned} \mu (S_I) \le \frac{1}{2} \sum _{i \notin I} \frac{1}{\ell _i} \qquad \text {and}\qquad \mu (S_{{\overline{I}}}) \le \sum _{i \in I} \frac{1}{\ell _i}. \end{aligned}$$

Consider the linear projection $\pi _I:{\mathbb {R}}^d \rightarrow {\mathbb {R}}^{I}$ vanishing on $S_I$. By Lemma 2.1,

$$\begin{aligned} \mu (S) \le \mu (S_I) + \mu (\pi _I(S)), \end{aligned}$$

so it only remains to show that

$$\begin{aligned} \mu (\pi _I(S)) \le \mu (S_{{\overline{I}}}). \end{aligned}$$

This holds because $\pi _I$ is an affine bijection from $S_{{\overline{I}}}$ to $\pi _I(S)$, so that $\pi _I(S)$ can be considered to be the same as $S_{{\overline{I}}}$ except regarded with respect to a (perhaps) finer lattice. $\square$

## Covering Minima of the Simplex $S(\omega )$

### The Covering Radius of $S(\omega )$

We here prove Theorem 1.4 and thus compute the covering radius of $S(\omega )={{\,\mathrm{conv}\,}}{\{{-}\omega _0 {\mathbf {1}}_d,\omega _1 e_1,\ldots ,\omega _d e_d\}}$.

#### Proof of Theorem 1.4

The simplex $S(\omega )$ can be triangulated into the $d+1$ simplices

$$\begin{aligned} S_i = {{\,\mathrm{conv}\,}}{(\{{\mathbf {0}},\omega _0 e_0, \omega _1 e_1, \ldots , \omega _d e_d\} \setminus \{\omega _i e_i\})}, \qquad 0 \le i \le d, \end{aligned}$$

where $e_0=-{\mathbf {1}}_d$. Writing $[d]_0 := \{0,1,\ldots ,d\}$, we define

$$\begin{aligned} \mathring{P}_i = \left\{ \sum _{j \in [d]_0 \setminus \{i\}} \alpha _j e_j : 0 \le \alpha _j < 1 \right\} , \end{aligned}$$

the half-open parallelotope spanned by the primitive edge directions of $S_i$ incident to the origin. Let $i\in [d]_0$ be fixed. Then, for any $x\in {\mathbb {R}}^d$ there is a lattice point $v_i\in {\mathbb {Z}}^d$ such that $x\in v_i+\lambda S_i$ and the dilation factor $\lambda \ge 0$ is the smallest possible. Let $L_i(x)$ be the set of all such lattice points $v_i$. For a fixed $v\in {\mathbb {Z}}^d$, we define

$$\begin{aligned} R_i(v) = \{x \in {\mathbb {R}}^d : v \in L_i(x)\} \end{aligned}$$

to be the region of points that are associated to *v* in this way.

Explicitly these regions are translates of the $\mathring{P}_i$, more precisely we claim that $R_i(v)=v+\mathring{P}_i$, for all $i\in [d_0]$. Indeed, let $x\in R_i(v)$, and let $\lambda \ge 0$ be smallest possible with $x\in v+\lambda S_i$. By the definition of $S_i$, we can write $x-v=\sum _{j\in [d]_0\setminus \{i\}}\alpha _je_j$, for some $\alpha _j\ge 0$. If there were an index *j* such that $\alpha _j\ge 1$, then $x\in v+e_j+\lambda S_i$ and the intersection of this simplex and $v+\lambda S_i$ would be a smaller homothetic copy of $S_i$ containing *x*. Thus, $\lambda$ would not be minimal and this contradiction implies that $x\in v+\mathring{P}_i$. Conversely, if $x-v=\sum _{j\in [d]_0\setminus \{i\}}\alpha _je_j\in \mathring{P}_i$, and $\lambda \ge 0$ is minimal such that $x\in v+\lambda S_i$, then $x-v$ lies in the facet of $\lambda S_i$ not containing the origin. Since $0\le \alpha _j<1$, for all $j\in [d]_0\setminus \{i\}$, the scalar $\lambda$ is not only minimal for *v*, but for any lattice point. Hence, $v \in L_i(x)$.

With this observation, the regions $R_i(v)$ are seen to be induced by the arrangement of the hyperplanes $\{x_i=a\}$, $\{x_i-x_j=a\}$ for all $j\in [d]_0\setminus \{i\}$ and $a\in {\mathbb {Z}}$, where we define $x_0=0$. We call this arrangement A$_d^i$. Moreover, for a point *x* in the interior of $R_i(v)$, the associated lattice point is unique, and we call it $v_i(x)$.

The smallest common refinement $\mathrm {A}_d$ of the arrangements $\mathrm {A}_d^0,\ldots ,\mathrm {A}_d^d$ is known as the *alcoved arrangement* (see [3, Chap. 7] for a detailed description). The full-dimensional cells of $\mathrm {A}_d$, also called its *chambers*, are lattice translations of the simplices

$$\begin{aligned} C_\pi ={{\,\mathrm{conv}\,}}{\bigl \{{\mathbf {0}}, e_{\pi (1)}, e_{\pi (1)}+e_{\pi (2)}, \ldots , e_{\pi (1)}+\ldots +e_{\pi (d)}\bigr \}}, \end{aligned}$$

where $\pi$ is a permutation of $\{1,\ldots ,d\}$. Each chamber of $\mathrm {A}_d$ is the intersection of regions $R_i(v)$. More precisely,

$$\begin{aligned} {{\,\mathrm{int}\,}}C_\pi&= R_0({\mathbf {0}}) \cap R_{\pi (1)}(e_{\pi (1)})\cap \ldots \cap R_{\pi (d)}(e_{\pi (1)} +\ldots +e_{\pi (d)}) \\&=\mathring{P}_0 \cap (e_{\pi (1)} + \mathring{P}_{\pi (1)} )\cap \ldots \cap (e_{\pi (1)} +\ldots +e_{\pi (d)} + \mathring{P}_{\pi (d)}). \end{aligned}$$

Therefore, the chambers $C_\pi$ are exactly those regions of points in ${\mathbb {R}}^d$ that, for each $i\in [d]_0$, are associated to the same lattice point, that is, $v_i(x)=v_i(y)$ for all $x,y\in {{\,\mathrm{int}\,}}C_\pi$.

After these preparations, we are ready to compute the covering radius of $S(\omega )$. Note that, since $[0,1]^d$ is a fundamental cell of ${\mathbb {Z}}^d$, we only need to find the smallest dilation factor $\mu$ so that the lattice translates of $\mu S(\omega )$ cover the unit cube. Moreover, we may focus on what happens within one chamber $C_\pi$, and by symmetry we assume that $\pi =\text {Id}$. Among all points in $C_\text {Id}={{\,\mathrm{conv}\,}}{\{{\mathbf {0}},e_1, e_1+e_2, \ldots ,e_1+\ldots +e_d\}}$, we are looking for a point *y* which is last covered by dilations of $S_i+e_{[i]}$, for some $i\in [d]_0$, and the factor of dilation needed. Here, we write $e_{[i]}=e_1+\ldots +e_i$. If we let $\ell _i:{\mathbb {R}}^d\rightarrow {\mathbb {R}}$ be the linear functional which takes value 1 on the facet $F_i$ of $S(\omega )$ that is opposite to $\omega _i e_i$, this is equivalent to

$$\begin{aligned} y=\mathop {\text {arg max}}\limits _{x \in C_\text {Id}} \min _{i \in [d]_0} |\ell _i(x-e_{[i]})|. \end{aligned}$$

The key observation is that *y* is the point where all the values $|\ell _i(y-e_{[i]})|$, $0 \le i \le d$, are equal. This is because $\ell _i(x-e_{[i]})$ is nonnegative for $x \in C_\text {Id}$ and because there is a positive linear dependence among the functionals $\ell _i$, so there cannot be a point $y'$ where they all achieve a larger value than at a point where they all achieve the same value. Therefore, *y* satisfies the conditions

$$\begin{aligned} \ell _0(y)=\ell _i(y-e_{[i]})\ \quad \text {for every }1\le i\le d. \end{aligned}$$

The explicit expression of the functionals $\ell _i$ is

$$\begin{aligned} \ell _0(x)=\sum _{j=1}^d \omega _j^{-1} x_j \quad \ \text {and}\ \quad \ell _i(x)\,=\sum _{j\in [d]\setminus \{i\}}\omega _j^{-1}x_j-\left( \,\sum _{j\in [d]_0\setminus \{i\}}\omega _j^{-1} \right) x_i. \end{aligned}$$

Thus we need to solve the following system of equations:

$$\begin{aligned} \sum _{j=1}^d \omega _j^{-1} y_j \, = \sum _{j \in [d] \setminus \{i\}} \omega _j^{-1}y_j -\left( \,\sum _{j \in [d]_0\setminus \{i\}}\omega _j^{-1}\right) y_i + \omega _0^{-1} + \sum _{j>i}\omega _j^{-1}, \end{aligned}$$

$1\le i\le d$. This system is solved by $y=(y_1,\ldots ,y_d)$ with

$$\begin{aligned} y_i = \frac{\omega _0^{-1} + \omega _{i+1}^{-1} + \ldots +\omega _d^{-1}}{\omega _0^{-1} + \omega _1^{-1} + \ldots +\omega _d^{-1}}. \end{aligned}$$

The value that the functionals take at *y* is by what we said above the covering radius of $S(\omega )$, and it is given by

$$\begin{aligned} \mu (S(\omega )) = \ell _0(y) = \frac{\sum _{0 \le i < j \le d} \omega _i^{-1}\omega _j^{-1}}{\sum _{i=0}^d \omega _i^{-1}}, \end{aligned}$$

as desired. $$\square $$

#### Corollary 5.1

Let $S \subseteq {\mathbb {R}}^d$ be a simplex with the origin in its interior and with rational vertex directions. If the primitive vertex directions $p_0,p_1,\ldots ,p_d$ of *S* satisfy $p_0+p_1+\ldots +p_d={\mathbf {0}}$, then Conjecture C holds for *S*.

#### Proof

The proof is basically given already in Corollary 4.11. Consider the lattice $\Lambda$ generated by $p_0,p_1,\ldots ,p_d$, and let *A* be the linear transformation sending $e_i$ to $p_i$, for $i=1,\ldots ,d$. Then, $\Lambda =A{\mathbb {Z}}^d$ and $S=AS(\omega )$ for a suitable $\omega \in {\mathbb {R}}^{d+1}_{>0}$. Since the $p_i$s are primitive, the lattice lengths $\ell _i=({{{\,\mathrm{Vol}\,}}_{{\mathbb {Z}}^d}S})/({{{\,\mathrm{Vol}\,}}_{\pi _i({\mathbb {Z}}^d)}(\pi _i(S))})$ are the same for every pair $(S,{\mathbb {Z}}^d)$, $(S,\Lambda )$, and $(S(\omega ),{\mathbb {Z}}^d)$. Using that $\Lambda \subseteq {\mathbb {Z}}^d$ is a sublattice, we therefore apply Theorem 1.4 and get

$$\begin{aligned} \qquad \qquad \qquad \qquad \qquad \mu (S) \le \mu (S,\Lambda ) = \mu (S(\omega ),{\mathbb {Z}}^d) = \frac{1}{2} \sum _{i=0}^d \frac{1}{\ell _i}. \qquad \qquad \qquad \qquad \,\square \end{aligned}$$

Observe that Theorem 1.4 says that (4) in Conjecture C is an equality for simplices of the form $S(\omega )$. Other simplices may also produce an equality, as the triangle $T = S({\mathbf {1}}_2) \oplus S'({\mathbf {1}}_2)$ shows:

$$\begin{aligned} \frac{1}{2}\cdot \frac{\sum _{i=0}^2 {{\,\mathrm{Vol}\,}}_{(\pi _i({\mathbb {Z}}^2)}(\pi _i(T))}{{{\,\mathrm{Vol}\,}}_{{\mathbb {Z}}^2}T} =\frac{1}{2} \cdot \frac{3 + 3 + 2}{4} = 1 = \mu (T). \end{aligned}$$

### The Covering Product Conjecture

The following conjecture was proposed in [10], which was the initial motivation to compute the covering minima of the simplex $S({\mathbf {1}}_{d+1})$.

#### Conjecture F

([10, Conj. 4.8])  For every convex body $K \subseteq {\mathbb {R}}^d$,

$$\begin{aligned} \mu _1(K)\cdot \ldots \cdot \mu _d(K)\cdot {{\,\mathrm{vol}\,}}K\ge \frac{d+1}{2^d}. \end{aligned}$$

Equality is attained for the simplex $S({\mathbf {1}}_{d+1})$.

Conjecture F is known to hold for $d=2$ [25]. We show it in arbitrary dimension for the simplices $S(\omega )$.

#### Corollary 5.2

For every $\omega \in {\mathbb {R}}^{d+1}_{>0}$, we have

$$\begin{aligned} \mu _1(S(\omega )) \cdot \ldots \cdot \mu _d(S(\omega )) \cdot {{\,\mathrm{Vol}\,}}_{{\mathbb {Z}}^d}(S(\omega ))\ge \frac{(d+1)!}{2^d}. \end{aligned}$$

Equality can hold only if $\omega _0=\omega _1=\ldots =\omega _d$.

#### Proof

Since every permutation of the vertices of $S({\mathbf {1}})$ is a unimodular transformation, and since the considered product functional is invariant under unimodular transformations, we can assume that $\omega _0\le \omega _1\le \ldots \le \omega _d$. By Theorem 1.4, the covering radius of $S(\omega )$ is given by

$$\begin{aligned} \mu (S(\omega )) = \frac{\sigma _{d-1}(\omega _0,\omega _1,\ldots ,\omega _d)}{\sigma _d(\omega _0,\omega _1,\ldots ,\omega _d)}, \end{aligned}$$

where $\sigma _j(\omega _0,\omega _1,\ldots ,\omega _d)=\sum _{0\le i_1<\ldots <i_j\le d}\prod _{\ell =1}^j\omega _{i_\ell }$ is the *j*-*th elementary symmetric function* in the $\omega _i$s. Writing $\omega _I=(\omega _0,\omega _{i_1},\ldots ,\omega _{i_j})$, for every index set $I=\{i_1,\ldots ,i_j\}\subseteq \{1,\ldots ,d\}$, $|I|=j$, we project onto the *j*-dimensional coordinate plane indexed by *I* and obtain $\mu _j(S(\omega ))\ge \mu _j(S(\omega _I))$. In particular, choosing $I=\{1,\ldots ,j\}$, we have

13 $$\begin{aligned} \mu _j(S(\omega )) \ge \frac{\sigma _{j-1}(\omega _0,\omega _1,\ldots ,\omega _j)}{\sigma _j(\omega _0,\omega _1,\ldots ,\omega _j)}. \end{aligned}$$

Next, in view of $\omega _j \ge \omega _{j-1} \ge \ldots \ge \omega _0$, we get

14 $$\begin{aligned} \begin{aligned} \frac{\sigma _{j-1}(\omega _0,\ldots ,\omega _j)}{\sigma _{j-1}(\omega _0,\ldots ,\omega _{j-1})}&=\frac{\sigma _{j-1}(\omega _0,\ldots ,\omega _{j-1})+\omega _j\sigma _{j-2}(\omega _0,\ldots ,\omega _{j-1})}{\sigma _{j-1}(\omega _0,\ldots ,\omega _{j-1})} \\&=1+\frac{\omega _j\sigma _{j-2}(\omega _0,\ldots ,\omega _{j-1})}{\sigma _{j-1}(\omega _0,\ldots ,\omega _{j-1})}\ge 1+\frac{\left( {\begin{array}{c}j\\ 2\end{array}}\right) }{j}=\frac{j+1}{2}, \end{aligned} \end{aligned}$$

with strict inequality unless $\omega _j = \omega _{j-1} = \ldots = \omega _0$.

Finally, computing the volumes of the pyramids over the $d+1$ facets of $S(\omega )$ with apex at the origin, we obtain ${{\,\mathrm{Vol}\,}}_{{\mathbb {Z}}^d}S(\omega )=\sigma _d(\omega _0,\omega _1,\ldots ,\omega _d)$. Combining this with (13) and (14) yields

$$\begin{aligned} \mu _1(S(\omega ))\cdot \ldots \cdot \mu _d(S(\omega ))\cdot {{\,\mathrm{Vol}\,}}_{{\mathbb {Z}}^d}(S(\omega ))&\ge \prod _{j=1}^d\frac{\sigma _{j-1}(\omega _0,\ldots ,\omega _j)}{\sigma _j(\omega _0,\ldots ,\omega _j)}\sigma _d(\omega _0,\ldots ,\omega _d)\\&= \prod _{j=1}^{d} \frac{\sigma _{j-1}(\omega _0,\ldots ,\omega _{j})}{\sigma _{j-1}(\omega _0,\ldots ,\omega _{j-1})} \ge \frac{(d+1)!}{2^d}. \end{aligned}$$

Furthermore, equality can only hold if $\omega _0 = \omega _1 = \ldots = \omega _d$ as otherwise (14) would be strict for $j=d$. $\square$

Note that if Conjecture B holds, then the simplex $S({\mathbf {1}}_{d+1})$ attains equality in Corollary 5.2 (this was the original motivation in [10] to state Conjecture B).

With the notation of the proof above, for each $I \subseteq \{1,\ldots ,d\}$, $|I|=j$, we have $\mu _j(S(\omega _I))\le \mu _j(S(\omega _0,\omega _1,\ldots ,\omega _j))$, just because $S(\omega )\subseteq S({\bar{\omega }})$, whenever $\omega _i\le {\bar{\omega }}_i$, for all *i*. Therefore, the bound in (13) is maximal among coordinate projections of $S(\omega )$. This suggests the following common generalization of Conjecture B and Theorem 1.4.

#### Conjecture 5.3

For every $\omega \in {\mathbb {R}}^{d+1}_{>0}$ with $\omega _0 \le \omega _1 \le \ldots \le \omega _d$, and every $j \in \{1,\ldots ,d\}$, the *j*-th covering minimum of the simplex $S(\omega )$ is attained by the projection to the first *j* coordinates. That is:

$$\begin{aligned} \mu _j(S(\omega )) = \mu _j(S(\omega _0,\dots , \omega _j)) = \frac{\sigma _{j-1}(\omega _0,\omega _1,\ldots ,\omega _j)}{\sigma _j(\omega _0,\omega _1,\ldots ,\omega _j)}. \end{aligned}$$

Besides the case $j=d$ (Theorem 1.4) also the case $j=1$ of Conjecture 5.3 holds. Assuming that $\omega _0\le \omega _1\le \ldots \le \omega _d$, it states that $\mu _1(S(\omega ))=1/({\omega _0+\omega _1})$. Since (13) provides the lower bound, this is equivalent to

$$\begin{aligned} \det ({\mathbb {Z}}^d | L_z) \le \frac{\Vert S(\omega )| L_z\Vert }{\omega _0+\omega _1}, \end{aligned}$$

for all primitive $z\in {\mathbb {Z}}^d\setminus \{{\mathbf {0}}\}$, where $L_z = {{\,\mathrm{lin}\,}}{\{z\}}$. In view of $\det ({\mathbb {Z}}^d| L_z) = \Vert z\Vert ^{-1}$ and $e_i| L_z = ({z_i}/{\Vert z\Vert ^2}) z$, it follows from an elementary computation.

## Conjecture D: Lattice Polytopes with *k* Interior Lattice Points

This section is devoted to prove Conjecture D in dimension two. The conjectured maximum covering radius $({d-1})/2+1/({k+1})$ is attained, in arbitrary dimension, by the polytopes of the form

$$\begin{aligned}{}[0,k+1] \oplus T_1\oplus \dots \oplus T_m, \end{aligned}$$

where each $T_i$ is a non-hollow lattice $d_i$-polytope of covering radius $d_i/2$, with $\sum _{i=1}^md_i=d-1$. The different $T_i$ can be translated to have their (unique) interior lattice point at different positions along the segment $[0,k+1]$ in much the same way as in the examples of Lemma 3.83.8. In the following we analyze the possibilities in dimensions two and three:

### Example 6.1

In dimension two we have a single $T_i$, the segment $[-1,1]$, but we can place it at different heights with respect to $[0,k+1]$. For each *k* we can construct $\lfloor (k+3)/2\rfloor$ non-isomorphic lattice polygons with *k* interior lattice points and of covering radius $1/2+1/({k+1})$, namely:

$$\begin{aligned} {{\,\mathrm{conv}\,}}{\{(0,0), (0,k), (-1,i), (1,i)\}},\ \quad i=0,\dots ,\lfloor (k+1)/2\rfloor . \end{aligned}$$

The case $i=0$ coincides with the triangle $M_k(0)$; the cases $i>0$ produce kite-shaped quadrilaterals. Observe that the triangle $M_k(1)\cong S(k,1,1)$ is very similar to $M_k(0)$ but has smaller area. One could expect it to achieve a larger covering radius but it does not, as computed in Remark 3.4:

$$\begin{aligned} \mu (M_k(1)) = \frac{k+2}{2k+1} < \frac{k+3}{2k+2} = \frac{1}{2} + \frac{1}{k+1} \quad \text { if } k > 1. \end{aligned}$$

### Example 6.2

In dimension three we can have $[0,k+1]\oplus T$ with $\dim T=2$ or $[0,k+1]\oplus T_1\oplus T_2$ with $\dim T_1=\dim T_2=1$. If the latter happens then $T_1=T_2=[-1,1]=I$ and, again, they can be placed at different heights along the segment $[0,k+1]$. Depending on whether $T_1$ and $T_2$ intersect $[0,k+1]$, in the interior or at an end-point, this gives quadratically many octahedra or linearly many triangular bipyramids, plus the square pyramid $[0,k+1] \oplus (I \oplus I)$ and the tetrahedron $M_k(0,0)$. In the case $[0,k+1]\oplus T$, *T* can be either $S({\mathbf {1}}_3)$ or $I\oplus I'$; the case $T=I \oplus I$ being already covered above. This produces two tetrahedra $[0,k+1]\oplus S({\mathbf {1}}_3)$ and $[0,k+1] \oplus I \oplus I'$, plus linearly many triangular bipyramids. As happened in dimension two, the computations of Remark 3.12 show that $M_k(1,0)$ and $M_k(1,1)$ have covering radius strictly smaller than $1+1/({k+1})$, even if their volume is smaller than that of $M_k(0,0)$.

Since Conjecture A holds in the plane (Corollary 3.6), to prove Conjecture D in dimension two it suffices to consider lattice polygons with at least two interior lattice points. More precisely, we show

### Theorem 6.3

Let *P* be a non-hollow lattice polygon with $k\ge 2$ interior lattice points. Then $\mu (P)\le 1/2+1/({k+1})$, with equality if and only if *P* is the direct sum of two lattice segments of lengths 2 and $k+1$.

Remember that a lattice polytope *P* has (*lattice*) *width* $\omega \in {\mathbb {N}}$ if there is an affine integer projection from *P* to the segment $[0,\omega ]$ but not to $[0,{\omega -1}]$. Equivalently, the width is the reciprocal of the first covering minimum. Every non-hollow lattice polytope has width at least two. Our next two lemmas deal with the case of width exactly two.

### Lemma 6.4

For a non-hollow lattice polygon *P* the following are equivalent:

(i) *P* has width equal to two.
(ii) The interior lattice points of *P* are collinear.

### Proof

The fact that width two implies that all interior lattice points are collinear is straightforward to check. For the converse, without loss of generality assume that the *k* interior lattice points of *P* are $(0,1),\dots ,(0,k)$, with $k\ge 2$. We claim that $P\subset [-1,1]\times {\mathbb {R}}$, which implies that *P* has width two with respect to the first coordinate. Suppose to the contrary that *P* has a lattice point (*x*, *y*) with $|x|\ge 2$. Then the triangle with vertices (0, 1), (0, 2), and (*x*, *y*) is not unimodular, which implies that it contains at least one lattice point other than its vertices, by Pick’s formula (cf. [3, Chap. 1.4]). That point is necessarily in ${{\,\mathrm{int}\,}}P$ and not on the line containing (0, 1) and (0, 2), a contradiction. $\square$

### Lemma 6.5

Theorem 6.3 holds if *P* has width two.

### Proof

We keep the convention from the previous proof that the interior lattice points in *P* are given by $(0,1),\dots ,(0,k)$, which implies that $P\subset [-1,1]\times [0,k+1]$. Let *S* be the segment $P\cap (\{0\}\times {\mathbb {R}})$, which contains all the interior lattice points. Observe that one endpoint of *S* is either (0, 0) or (0, 1/2) and the other is either $(0,k+1)$ or $(0,k+1/2)$. We distinguish three cases, depending on whether none, one, or both of them are lattice points:

- If exactly one endpoint is a lattice point, then *P* contains a copy of $M_k(1)$, whose covering radius is strictly smaller than $1/2+1/({k+1})$ (see Example 6.1).
- If no endpoint is a lattice point, then $S=\{0\}\times [1/2,k+1/2]$ and *P* is the convex hull of its two edges containing the endpoints of *S*. Without loss of generality we assume
  $$\begin{aligned} P= {{\,\mathrm{conv}\,}}{\{(-1,0), (1,1), (-1,a), (1, 1+b)\}}, \end{aligned}$$
  where *a* and *b* are nonnegative integers with $a+b=2k$. There are two possibilities: If $a=b=k$, then *P* is a parallelogram of covering radius at most 1/2, because $(1/2) P$ contains a fundamental domain of ${\mathbb {Z}}^2$. If $a\ne b$, then one of them, say *a*, is at least $k+1$. In this case, *P* contains the triangle ${{\,\mathrm{conv}\,}}{\{(-1,0),(-1,a),(1,1)\}}$ whose covering radius is bounded by $1/2+1/a\le 1/2+1/(k+1)$. Since triangles are tight by Lemma 2.7, equality can only hold when *P* coincides with this triangle, implying $b=0$. But in that case $a=2k$ and $1/2+1/a<1/2+1/(k+1)$, since $k\ge 2$.
- If both endpoints of *S* are lattice points, then they are given by (0, 0) and $(0,k+1)$. Applying Lemma 2.1 to the projection that forgets the second coordinate gives the upper bound: The fiber *S* has length $k+1$ and the projection of *P* has length 2. For the case of equality, observe that if *P* has lattice points $u\in \{-1\}\times {\mathbb {R}}$ and $v\in \{1\}\times {\mathbb {R}}$ such that the mid-point of *uv* is integral, then *P* contains (an affine image of) the direct sum of $[-1,1]$ and a segment of length $k+1$. Since that direct sum is tight by Lemma 2.8, *P* either is given by this direct sum or it has strictly smaller covering radius.
  Thus, we can assume that *P* does not have such points *u* and *v*. This implies that *P* has a single lattice point on each side of *S*. Without loss of generality we can assume
  $$\begin{aligned} P= {{\,\mathrm{conv}\,}}{\{(0,0), (0,k+1), (-1,0), (1,a)\}}, \end{aligned}$$
  for an odd $a\in [1,2k+1]$. We claim that the *proof* of Lemma 2.1 implies that $\mu (P)$ is *strictly smaller* than $\lambda :=1/2 + 1/(k+1)$. Indeed, that proof is based on the fact that $\lambda P$ contains the following parallelogram *Q*, which is a fundamental domain for ${\mathbb {Z}}^2$:
  $$\begin{aligned} Q={{\,\mathrm{conv}\,}}{\{({-}1/2,0),({-}1/2,1),(1/2,a/2),(1/2,1+a/2)\}}. \end{aligned}$$
  But we can argue that, moreover, the vertices of *Q* are its only points not contained in the interior of $\lambda P$, and that each of these vertices is in the interior of some lattice translation of $\lambda P$ because the vertical offset of the left and right edges of *Q* is not an integer. This implies $\lambda$ is strictly larger than $\mu (P)$.$\square$

For the rest of the proof of Theorem 6.3 we can assume $\omega \ge 3$. Let *m* be the maximum number of collinear lattice points in our polygon *P*. Applying Lemma 2.1 to the projection along the line containing those *m* points gives

15 $$\begin{aligned} \mu (P) \le \frac{1}{\omega }+ \frac{1}{m-1}. \end{aligned}$$

Another useful fact is that along the direction that attains the width $\omega$ there are $\omega -1$ parallel lines intersecting the interior of *P*, each of them contains at most *m* lattice points, and with every lattice point of *P* lying on one of those lines. Thus

16 $$\begin{aligned} k \le (\omega -1) m. \end{aligned}$$

These two bounds are enough to show that

### Lemma 6.6

Theorem 6.3 holds if $m\ge 4$, except perhaps for $(\omega ,m) = (3,4)$.

### Proof

By (15), the statement is trivial unless

$$\begin{aligned} \frac{1}{\omega }+ \frac{1}{m-1} >\frac{1}{2}. \end{aligned}$$

There are five integer solutions of this equation with $\omega \ge 3$ and $m-1\ge 3$:

$$\begin{aligned} (\omega ,m-1)\in \{(3,3), (3,4), (4,3), (3,5), (5,3)\}. \end{aligned}$$

We only need to look at the last four:

- If $(\omega ,m-1)\in \{(3,5),(5,3)\}$ then (15) gives $\mu (P)\le 1/3+1/5=1/2+1/30$. This is smaller than $1/2+1/(k+1)$, because (16) gives, respectively, $k\le 12$ and $k\le 16$.
- If $(\omega ,m-1)\in \{(3,4),(4,3)\}$ then $\mu (P)\le 1/3+1/4=1/2+1/12$. For (3, 4) this is enough since (16) gives $k\le 10$. For (4, 3), however, (16) gives $k\le 12$, so we still need to consider the cases $k=11$ or 12. For these we use the following argument: $\omega =4$ implies that, along the direction where $\omega$ is attained, we have three intermediate lattice lines intersecting *P*. Along these lines we have to place our $k\ge 11$ points, and no more than four on each line (because $m=4$). Thus, each line gets at least three points. This makes *P* contain a parallelogram *Q* with two parallel edges of lattice length two and of width two with respect to the direction of those edges. We have that *Q* is a fundamental domain of $(2{\mathbb {Z}})^2$, which implies $\mu (P)\le \mu (Q)\le 1/2$. $\square$

**Fig. 3:**
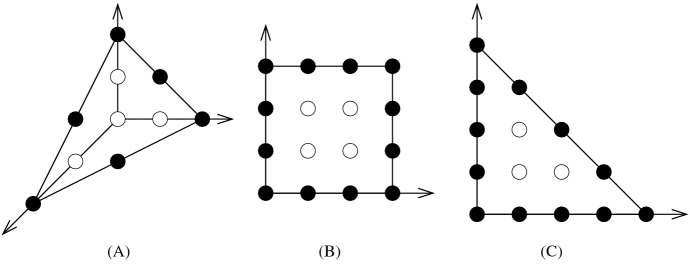
The three possibilities in the case $m=3$

Thus, the cases that remain are $m\le 3$ or $(\omega ,m)=(3,4)$. These can be proven with a case study that we only sketch here. The details can be found in the arXiv version of this article [7]. The case study goes as follows:

- For the case $(w,m) = (3,4)$, in [7, Lem. 6.7] we show that one of the following three things happen:
  - $k\le 5$, in which case $\mu (P) \le 1/3 + 1/3 \le 1/2 + 1/(k+1)$.
  - *P* contains a fundamental domain *Q* of $(2{\mathbb {Z}})^2$. As in the last paragraph of the previous proof, this implies $\mu (P)\le \mu (Q)\le 1/2$.
  - *P* has four collinear lattice points along one of the two intermediate lines in the direction attaining the width, and (at least) three of them are interior to *P*. In this case the intersection of *P* with that line has length at least $3+1/3=10/3$, so (15) can be strengthened to
    $$\begin{aligned} \mu (P) \le \frac{1}{3} + \frac{3}{10}= \frac{19}{30} < \frac{1}{2} + \frac{1}{7}. \end{aligned}$$
    This gives the statement if $k\in \{5,6\}$. In the case $k\ge 7$ we must have four collinear lattice points in one of the two intermediate lines, so that we can further improve (15) using 11/3 for the length. Then
    $$\begin{aligned} \mu (P) \le \frac{1}{3} + \frac{3}{11}=\frac{20}{33}<\frac{1}{2}+\frac{1}{9}. \end{aligned}$$
    This is enough since $(w,m) = (3,4)$ implies $k\le 8$, by (16).
- The case $m\le 2$ is trivial: it implies that *P* does not have two lattice points in the same class modulo $2{\mathbb {Z}}\times 2{\mathbb {Z}}$, so it has at most four lattice points. The only non-hollow lattice polygon with at most four lattice points is $S({\mathbf {1}}_3)$.
- For the case $m=3$, in [7, Lemma 6.8] we show that $\omega \ge 3$ and $m=3$ imply that *P* cannot have three collinear interior lattice points. Since the interior lattice points cannot all be collinear (by Lemma 6.4), they must form either a unimodular triangle, a unit parallelogram, or $S({\mathbf {1}}_3)$. Thus, *P* is contained in one of the three polygons of Fig. 3. From there, ad-hoc arguments show that always $\mu (P) \le 1/2 + 1/(k+1)$.

### Remark 6.7

Lattice polygons with $m \le 3$ contain at most nine lattice points in total, since they cannot have two points in the same residue class modulo $(3{\mathbb {Z}})^2$. In particular, they have $k\le 6$. On the other hand, the polytopes with $(\omega ,m)=(3,4)$ have $k\le 8$ by (16). Thus, the cases not covered by Lemmas 6.5 and 6.6 have between three and eight interior lattice points. Castryck [6] enumerated all lattice polygons with $k\le 30$ up to unimodular equivalence, and showed that there are $120+211+403+714+1023+1830$ of them with *k* equal to 3, 4, 5, 6, 7, and 8. Hence, the arguments sketched above can be replaced by a computer-aided computation of the covering radius of these 4301 polygons. (In fact, the covering radius needs only to be computed for those with $m\in \{3,4\}$).

## Figures and Tables

**Fig. 4:**
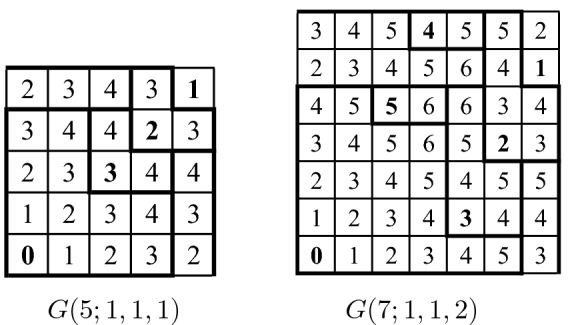
Graphical computation of $\delta (G(5;1,1,1))=4$ and $\delta (G(7;1,1,2))=6$, implying that $\mu (T(1,1,1,2))=7/5$ and $\mu (T(1,1,2,3))=9/7$

**Fig. 5:**
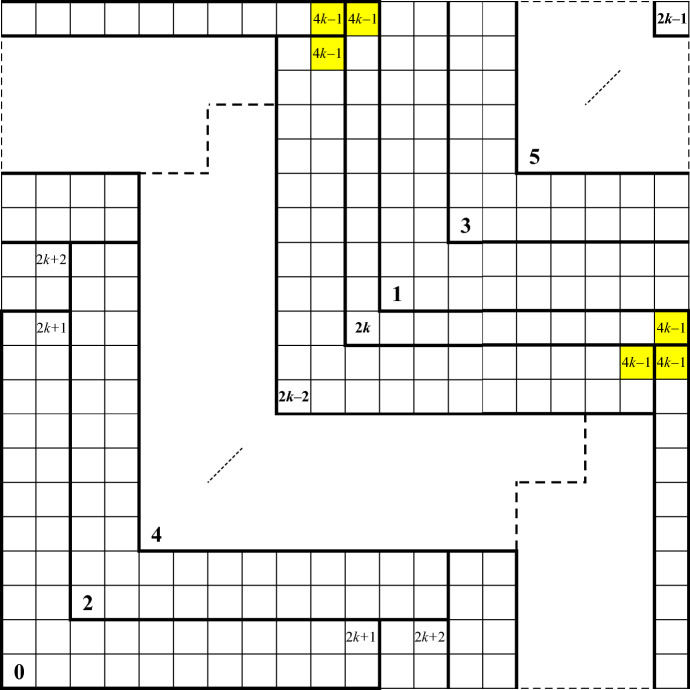
Computation of $\delta (G(4k;1,2k-1,2k-1))$, implying $\mu (M_k(1,1))=1+1/2k$. Only the distance to some cells is shown. Cells achieving the diameter are highlighted

## Appendix A: The Covering Radius via a Mixed-Integer Program

Here we describe an algorithmic approach to the computation of covering radii based on a formulation of $\mu (P)$ as the optimal value of a mixed-integer program. This formulation is already implicit in Kannan’s paper [16, Sect. 5].

Let $P=\{x\in {\mathbb {R}}^d:a_i^\intercal x\le b_i,\,1\le i\le m\}$ be a polytope with outer facet normals $a_i\in {\mathbb {R}}^d$ and right hand sides $b_i\in {\mathbb {R}}$. Without loss of generality, we assume that $b_i>0$, that is, *P* contains the origin in its interior. Since *P* is bounded, there exists a finite subset $N_P \subseteq {\mathbb {Z}}^d$ such that $\mu (P) P+N_P$ contains the unit cube $[0,1]^d$.

### Proposition A.1

The covering radius $\mu (P)$ is equal to the optimal value of the following linear mixed-integer program:

$$\begin{aligned}&maximize&\mu&\\&s.t.&a_i^\intercal x&\ge \mu b_i+a_i^\intercal \ell -M(1-y_i^\ell ),\quad&\forall i=1,\ldots ,m, \ \forall \ell \in N_P,\\&\sum _{i=1}^{m} y_i^\ell&\ge 1,&\forall \ell \in N_P, \\&y_i^\ell&\in \{0,1\},&\forall i=1,\ldots ,m, \ \forall \ell \in N_P, \\&x&\in [0,1]^d.&\end{aligned}$$

The constant $M > 0$ is chosen large enough such that every non-active inequality involving *M* is redundant.

### Proof

By the periodicity of the arrangement $\mu P + {\mathbb {Z}}^d$, we get that

$$\begin{aligned} \mu (P)=\min {\{\mu \ge 0:[0,1]^d\subseteq \mu P+N_P\}}. \end{aligned}$$

Hence, the covering radius equals the minimal $\mu \ge 0$ such that for all $x\in [0,1]^d$ there exists an $\ell \in N_P$ such that $x\in \mu P+\ell$. This gives a mixed-integer program with infinitely many constraints. In order to turn it into a finite program, we may also interpret the covering radius as the supremum among $\mu \ge 0$ such that there exists an $x\in [0,1]^d$ such that $x\notin \mu P+N_P$.

Modeling this non-containment condition can be done as follows: For a fixed $\ell \in N_P$, we have $x\notin \mu P+\ell$ if and only if there exists a defining inequality of *P* that is violated, that is, there exists an $i\in \{1,\ldots ,m\}$ such that $a_i^\intercal x>\mu b_i+a_i^\intercal \ell$. Introducing the binary variable $y_i^\ell$ for each $1\le i\le m$ and each $\ell \in N_P$, and using a large enough constant $M>0$, this is modeled by the first two lines in the program, as the condition $\sum _{i=1}^my_i^\ell \ge 1$ ensures that at least one inequality is violated for $\ell$.

We can replace the supremum by a maximum and the strict inequality $a_i^\intercal x > \mu b_i + a_i^\intercal \ell$ by a non-strict one, since *P* is compact and the covering radius is in fact an attained maximum. $\square$

In order to make this formulation effective, we need to find a suitable finite subset $N_P\subseteq {\mathbb {Z}}^d$: A point $x\in [0,1]^d$ is contained in $z+\mu (P) P$, for some $z\in {\mathbb {Z}}^d$, if and only if $z\in [0,1]^d-\mu (P) P$. Hence, for any theoretically proven upper bound $\mu (P)\le \mu$, we can solve the mixed-integer program in Proposition A.1 with respect to $N_P=([0,1]^d-\mu P)\cap {\mathbb {Z}}^d$ and obtain the covering radius of *P*.

## Appendix B: Graphical Method for Covering Radii of Simplices

Let $T={{\,\mathrm{conv}\,}}{\{v_0,v_1,\ldots ,v_d\}}$ be a lattice simplex of normalized volume $V={{\,\mathrm{Vol}\,}}_\Lambda T$ with respect to a certain lattice $\Lambda$. The affine map defined by $v_0\mapsto {\mathbf {0}}$ and $v_i \mapsto V \cdot e_i$, $i=1,\dots ,d$, sends *T* to the dilated standard simplex $V\cdot {{\,\mathrm{conv}\,}}{\{{\mathbf {0}},e_1,\dots ,e_d\}}$ and $\Lambda$ to an intermediate lattice between $V{\mathbb {Z}}^d$ and ${\mathbb {Z}}^d$, which we still denote by $\Lambda$. Observe that $\Lambda _T:= \Lambda / V{\mathbb {Z}}^d$ is a subgroup of ${\mathbb {Z}}^d/ V{\mathbb {Z}}^d = ({\mathbb {Z}}_V)^d$ of order *V* and that

$$\begin{aligned} {\mathbb {Z}}^d / \Lambda = ({\mathbb {Z}}_V)^d / (\Lambda / V{\mathbb {Z}}^d) \end{aligned}$$

is, hence, a finite abelian group of order $V^{d-1}$. The *Cayley digraph* *G* associated with the quotient group ${\mathbb {Z}}^d/\Lambda$ is defined as the directed graph with vertex set ${\mathbb {Z}}^d/\Lambda$ and edges $(x+\Lambda ,x+e_i+\Lambda )$, for $x\in {\mathbb {Z}}^d$ and $1\le i\le d$. The following is a particular case of [21, Lems. 3 & 4] (cf. also [10, Thm. 4.11]):

### Lemma B.1

In these conditions, let $\delta (G)$ be the (directed) diameter of *G*. That is, $\delta (G)$ is the maximum distance from ${\mathbf {0}}$ to any other node of *G*. Then

$$\begin{aligned} \mu (T) = \frac{\delta (G)+d}{V}. \end{aligned}$$

### Proof

The covering radius of the standard *d*-simplex ${{\,\mathrm{conv}\,}}{\{{\mathbf {0}},e_1,\dots ,e_d\}}$ with respect to the sublattice $\Lambda$ of ${\mathbb {Z}}^d$ equals $\delta (G)+d$. (This is the case $v=(1,\ldots ,1)$ of [10, Thm. 4.11]). We divide this by *V* since we are looking at the *V*-th dilation of the standard simplex. $\square$

Let $p\in T$ be a lattice point in the lattice simplex $T={{\,\mathrm{conv}\,}}{\{v_0,\dots ,v_d\}}$ from above. For $i\in \{0,\dots ,d\}$, let $a_i$ be the normalized volume of the pyramid with apex *p* over the facet of *T* opposite to $v_i$. Observe that the normalized volume of *T* is given by $V=\sum _ia_i$. In fact, $(1/V)(a_0,\dots ,a_d)$ is the vector of barycentric coordinates of *p* in *T*.

### Lemma B.2

Assume $\gcd {(a_0,a_1,\dots ,a_d)}=1.$ Then *T* is equivalent to the simplex $V \cdot {{\,\mathrm{conv}\,}}{\{{\mathbf {0}},e_1,\dots ,e_d\}}$ with respect to the lattice $V{\mathbb {Z}}^d+(a_1,\dots ,a_d){\mathbb {Z}}$. In particular, the graph *G* of Lemma B.1 equals the Cayley digraph of $({\mathbb {Z}}_V)^d/{\langle (a_1,\dots ,a_d)\rangle }$ with respect to the standard generators.

### Proof

The affine map *f* sending $v_0$ to ${\mathbf {0}}$ and every other $v_i$ to $Ve_i$ fulfils $f(T)= V \cdot {{\,\mathrm{conv}\,}}{\{{\mathbf {0}},e_1,\dots ,e_d\}}$ and $f(p)=p':=(a_1,\dots ,a_d)$, since *p* has the same barycentric coordinates in *T* as $p'$ has in *f*(*T*). Also, $\Lambda ':=V{\mathbb {Z}}^d+(a_1,\dots ,a_d){\mathbb {Z}}$ is clearly a sublattice of $f(\Lambda )$, where $\Lambda$ is the ambient lattice of *T*. We only need to show that $\Lambda '=f(\Lambda )$ and for this it is enough to check that the normalized volume of *f*(*T*) with respect to $\Lambda '$ equals *V*.

This normalized volume is the order of the quotient $\Lambda '/V{\mathbb {Z}}^d$, and this quotient is a cyclic group generated by $p'+V{\mathbb {Z}}^d$. Thus, the normalized volume is the smallest $k\in {\mathbb {N}}$ such that $k(a_1,\dots ,a_d)\in V{\mathbb {Z}}^d$. We have $k=V$ since $V=a_0+\dots +a_d$ gives $\gcd {(V,a_1,\dots ,a_d)}=\gcd {(a_0,a_1,\dots ,a_d)}=1$. $\square$

Lemma B.2 implies that a lattice simplex is determined, modulo unimodular equivalence, by the *volume vector* $(a_0,\dots ,a_d)$ of any lattice point *p* in it, as long as $\gcd {(a_0,\dots ,a_d)}=1$. Since $\Lambda /V{\mathbb {Z}}^d$ is then cyclic with generator $p+V{\mathbb {Z}}^d$, we call *T* the *cyclic simplex generated by* $(a_0,\dots ,a_d)$. We denote it by $T(a_0,\dots ,a_d)$ and denote by $G(V;a_1,\dots ,a_d)$ the digraph in the statement.

In what follows we are interested in cyclic *tetrahedra* *T*(*a*, *b*, *c*, *d*). When $a=1$, Lemmas B.1 and B.2 are particularly easy to apply, since then *G*(*V*; *a*, *b*, *c*) coincides with the Cayley digraph of $({\mathbb {Z}}_V)^2$ with respect to the generators (1, 0), (0, 1), and $(-b,-c)$. That is, *G*(*V*; *a*, *b*, *c*) has $({\mathbb {Z}}_V)^2$ as vertex set and from each vertex (*i*, *j*) we have the following three arcs:

$$\begin{aligned} (i,j) \rightarrow (i,j+1), \quad (i,j) \rightarrow (i+1,j), \quad (i,j) \rightarrow (i-b,j-c). \end{aligned}$$

### Example B.3

Figure 4 shows the computation of the covering radii of *T*(1, 1, 1, 2) ($V=5$) and *T*(1, 1, 2, 3) ($V=7$): a grid with $V^2$ cells represents the nodes of *G*(5; 1, 1, 1) and *G*(7; 1, 1, 2) with the origin at the south-west corner. The grid has to be regarded as a torus, so that every cell has an east, west, north, and south neighbor. A step north or east increases the distance from the origin by one, unless the cell that we move to can be reached by a shorter path. When this happens, the corresponding arc of *G*(*V*; *a*, *b*, *c*) is not used in any shortest path from the origin, and we highlight in bold the corresponding wall between cells. Using this idea, one can compute the distance from the origin to each cell in a breadth-first search manner.

Observe that, by commutativity, we only need to consider paths that first use edges with step $(-b,-c)$ and then east or north steps. Thus, in order to verify that the distances we have put are correct in the whole diagram, only the distances along the path with steps $(-b,-c)$ starting at the origin need to be checked. The cells along that path have their distances also in bold, and they coincide with the cells with bold south and west walls. The path finishes when it arrives in a cell that can be more shortly reached from the origin by only east and north steps.

### Corollary B.4

$$\mu (T(1,1,1,2))=7/5$$ and $$\mu (T(1,1,2,3))=9/7$$.

This method can also be applied to the tetrahedra $M_k(1,1)$ of Sect. 3.2:

### Lemma B.5

For every $k\in {\mathbb {N}}$ we have

$$\begin{aligned} \mu (M_k(1,1)) = 1+ \frac{1}{2k}. \end{aligned}$$

### Proof

$M_k(1,1)$ has normalized volume 4*k* and the point $p=(0,0,1)$ has barycentric coordinates $(1/{4k})(1,1,2k-1,2k-1)$. Thus, $M_k(1,1) \cong T(1,1,2k-1,2k-1)$. Figure 5 shows that $\delta (G(4k;1,2k-1,2k-1))= 4k-1$, from which Lemmas B.1 and B.2 give $\mu (M_k(1,1)) = (4k+2)/4k$. $\square$

## Appendix C: The 26 Minimal Non-hollow Lattice 3-Polytopes; Proof of Theorem 3.9

The 26 minimal non-hollow lattice 3-polytopes with a single interior lattice point were classified by Kasprzyk [18]. We list them in Tables 1 and 2, in the same order as they appear in Kasprzyk’s Tables 2 and 4. Table 1 contains the 16 that are tetrahedra and Table 2 the 10 that are not.

Vertex coordinates are given as the columns of a matrix, and chosen so that the unique interior point is the origin. For the tetrahedral examples in Table 1 we include the *volume vector* (*a*, *b*, *c*, *d*), consisting of the normalized volumes of the pyramids from the origin over the facets. When the volume vector is primitive, our tetrahedron equals the cyclic tetrahedron *T*(*a*, *b*, *c*, *d*) of Lemma B.2. In some cases, an additional “description” of the example is given, which helps us later to bound its covering radius. For example, via this description we can identify the nine polytopes from Lemma 3.83.8 that have covering radius 3/2.

The exact covering radius, computed with the algorithm of Appendix AA using the SCIP solver in exact solving mode [8], is also shown. All except those of Lemma 3.83.8 have $\mu <3/2$, which provides a computer proof of Theorem 3.9. In the rest of this section we include a computer-free proof.

### C.1 The Sixteen Tetrahedra

**Table 1:** The 16 minimal non-hollow tetrahedra with exactly one interior lattice point, with their covering radii

$$\begin{pmatrix}-1 &{} 1 &{} 0 &{} 0 \\ -1 &{} 0 &{} 1 &{} 0 \\ -1 &{} 0 &{} 0 &{} 1\end{pmatrix}$$	$$\begin{pmatrix}-2 &{} 2 &{} 0 &{} 0 \\ -2 &{} 1 &{} 1 &{} 0 \\ -1 &{} 0 &{} 0 &{} 1\end{pmatrix}$$	$$\begin{pmatrix}-5 &{} 5 &{} 0 &{} 0 \\ -3 &{} 2 &{} 1 &{} 0 \\ -2 &{} 1 &{} 0 &{} 1\end{pmatrix}$$	$$\begin{pmatrix}-1 &{} 1 &{} 0 &{} 0 \\ -1 &{} 0 &{} 1 &{} 0 \\ -2 &{} 0 &{} 0 &{} 1\end{pmatrix}$$
(1, 1, 1, 1)	(2, 2, 2, 2)	(5, 5, 5, 5)	(1, 1, 1, 2)
$S({\mathbf {1}}_4)$	$(I \oplus I')'\oplus I$		
${\varvec{\mu }} = \mathbf{3}/\mathbf{2}$	${\varvec{\mu }} = \mathbf{3}/\mathbf{2}$	$\mu =9/10$	$\mu =7/5$
$$\begin{pmatrix}-1 &{} 1 &{} 0 &{} 0 \\ -1 &{} 0 &{} 1 &{} 0 \\ -3 &{} 0 &{} 0 &{} 1\end{pmatrix}$$	$$\begin{pmatrix}-1 &{} 1 &{} 0 &{} 0 \\ -2 &{} 0 &{} 1 &{} 0 \\ -2 &{} 0 &{} 0 &{} 1\end{pmatrix}$$	$$\begin{pmatrix}-1 &{} 1 &{} 0 &{} 0 \\ -2 &{} 0 &{} 1 &{} 0 \\ -3 &{} 0 &{} 0 &{} 1\end{pmatrix}$$	$$\begin{pmatrix}-1 &{} 1 &{} 0 &{} 0 \\ -2 &{} 0 &{} 1 &{} 0 \\ -4 &{} 0 &{} 0 &{} 1\end{pmatrix}$$
(1, 1, 1, 3)	(1, 1, 2, 2)	(1, 1, 2, 3)	(1, 1, 2, 4)
$S({\mathbf {1}}_3) \oplus I'$	$S({\mathbf {1}}_3)' \oplus I$		$(I \oplus I')^\circ \oplus I'$
${\varvec{\mu }} = \mathbf{3}/\mathbf{2}$	${\varvec{\mu }} = \mathbf{3}/\mathbf{2}$	$\mu =9/7$	${\varvec{\mu }} = \mathbf{3}/\mathbf{2}$
$$\begin{pmatrix}-1 &{} 1 &{} 0 &{} 0 \\ -3 &{} 0 &{} 1 &{} 0 \\ -4 &{} 0 &{} 0 &{} 1\end{pmatrix}$$	$$\begin{pmatrix}-1 &{} 1 &{} 0 &{} 0 \\ -3 &{} 0 &{} 1 &{} 0 \\ -5 &{} 0 &{} 0 &{} 1\end{pmatrix}$$	$$\begin{pmatrix}-1 &{} 1 &{} 0 &{} 0 \\ -4 &{} 0 &{} 1 &{} 0 \\ -6 &{} 0 &{} 0 &{} 1\end{pmatrix}$$	$$\begin{pmatrix}-2 &{} 1 &{} 0 &{} 0 \\ -3 &{} 0 &{} 1 &{} 0 \\ -5 &{} 0 &{} 0 &{} 1\end{pmatrix}$$
(1, 1, 3, 4)	(1, 1, 3, 5)	(1, 1, 4, 6)	(1, 2, 3, 5)
$\mu =11/9$	$\mu =13/10$	$\mu =4/3$	$\mu =12/11$
$$\begin{pmatrix}-3 &{} 1 &{} 0 &{} 0 \\ -4 &{} 0 &{} 1 &{} 0 \\ -5 &{} 0 &{} 0 &{} 1\end{pmatrix}$$	$$\begin{pmatrix}-1 &{} 1 &{} 0 &{} 0 \\ -3 &{} 0 &{} 2 &{} 0 \\ -4 &{} 0 &{} 1 &{} 1\end{pmatrix}$$	$$\begin{pmatrix}-3 &{} 2 &{} 0 &{} 0 \\ -4 &{} 1 &{} 1 &{} 0 \\ -5 &{} 1 &{} 0 &{} 1\end{pmatrix}$$	$$\begin{pmatrix}-4 &{} 3 &{} 0 &{} 0 \\ -3 &{} 1 &{} 1 &{} 0 \\ -5 &{} 2 &{} 0 &{} 1\end{pmatrix}$$
(1, 3, 4, 5)	(2, 2, 3, 5)	(2, 3, 5, 7)	(3, 4, 5, 7)
	$$Pyr _4(S({\mathbf {1}}_3))$$		
$\mu =14/13$	$\mu =7/6$	$\mu =1$	$\mu =18/19$

For most of the tetrahedra in Table 1 we are going to bound the covering radius based solely on the volume vector of its interior point. We need the following auxiliary result about the covering radius of some (perhaps non-lattice) triangles.

#### Proposition C.1

For each $v \in {\mathbb {R}}_{\ge 1}^2$, let $\Delta _v := {{\,\mathrm{conv}\,}}{\{-v,e_1,e_2\}}$. We have $\mu (\Delta _v) \le 1$. Equality holds if and only if $v \in \{(a,1),(1,a)\}$ with $1 \le a \le 2$.

#### Proof

Due to symmetry, we can assume $v = (v_1,v_2)$ with $v_1 \ge v_2$. If $v_2 > 1$, then $\Delta _v$ strictly contains the triangle $\Delta _w$, for some $w = (w_1,1)\in {\mathbb {R}}^2_{\ge 1}$. By Lemma 2.7 triangles are tight for every lattice, so that $\mu (\Delta _v) < \mu (\Delta _w)$ and it thus suffices to consider $v = (a,1)$, for $a \ge 1$.

Let $F_0$ be the edge of $\Delta _v$ not containing *v*, and let $F_1$ and $F_2$ be the edges of $\Delta _v$ not containing $e_1$ and $e_2$, respectively. Further, let $\ell = \{(x,y) : x+y=1\}$ be the line containing $F_0$. An elementary calculation gives

$$\begin{aligned} \ell \cap (F_1 + e_1)&= \biggl \{\biggl (\frac{2}{a+2},\frac{a}{a+2}\biggr )\biggr \}, \quad \ell \cap (F_2+e_2)=\biggl \{\biggl (\frac{1}{a+2},\frac{a+1}{a+2}\biggr )\biggr \}, \\ \ell \cap (F_1 + (1,1))&=\biggl \{\biggl (\frac{2-a}{a+2},\frac{2a}{a+2}\biggr )\biggr \}, \quad \ell \cap (F_2 + (1,1))=\biggl \{\biggl (\frac{2}{a+2},\frac{a}{a+2}\biggr )\biggr \}. \end{aligned}$$

This already shows that the translates $\{0,1\}^2 + \Delta _v$ cover the unit cube $[0,1]^2$, for every $a \ge 1$, so that $\mu (\Delta _v) \le 1$ as claimed.

In order to decide the equality case, observe that in the covering of $[0,1]^2$ by these four translates, the point $(2/(a+2),a/(a+2))$ is covered last, and is not contained in the interior of any of the four triangles. However, the translate $(2,1)+\Delta _v$ may contain this point in the interior. Noting that

$$\begin{aligned} \ell \cap (F_1 + (2,1)) = \biggl \{\biggl (\frac{4-a}{a+2},\frac{2a-2}{a+2}\biggr )\biggr \}, \end{aligned}$$

this happens if and only if $4-a < 2$, that is, $a > 2$. $\square$

#### Remark C.2

Every non-hollow lattice triangle is isomorphic to some $\Delta _v$ considered with respect to a superlattice of ${\mathbb {Z}}^2$. (Let (*a*, *b*, *c*) be the volume vector of an interior point, with $a\le b \le c$, and take $v=(b/a,c/a)$). With this, Proposition C.1 provides another proof of Conjecture A in the plane. This approach fails in higher dimensions since, for example, we have computed that the tetrahedron $\Delta _{(3/2,1,1)}$ has covering radius $14/9 >3/2$.

Let *T* be a lattice tetrahedron with the origin ${\mathbf {0}}$ in its interior and let $(a,b,c,d)\in {\mathbb {N}}^4$ be its volume vector, written with $a\le b \le c \le d$. Let *A*, *B*, *C*, *D* be the vertices of *T* labeled in the natural way (so that *a* is the determinant of *BCD*, etc.).

#### Lemma C.3

With this notation, suppose that the triangle *OCD* is unimodular. If either of the conditions (i) or (ii) below holds, then $\mu (T)<3/2$.

(i) $$a+b\le c$$ and $$(a,b,c) \ne (1,1,2)$$.
(ii) $$a+b\ge 4$$, $$3c\ge a+b+d$$, and $$(a,b,c,d)\ne (2,2,2,2)$$.

#### Proof

Since the triangle *OCD* is unimodular, there is no loss of generality in taking $C=(1,0,0)$ and $D=(0,1,0)$. Once this is done, *A* and *B* must have *z* coordinate equal to *b* and $-a$, in order for the determinants of *BCD* and *ACD* to be *a* and *b*, respectively. In order for the determinants of *ABD* and *ABC* to be *c* and *d*, the segment *AB* must intersect the plane $z=0$ at the point $(-c/(a+b),-d/(a+b))$. That is, $T\cap \{z=0\}$ is the triangle $\Delta _{(c/(a+b),d/(a+b))}$ of Proposition C.1. Then, Lemma 2.1 applied to projecting along the *z* coordinate gives

$$\begin{aligned} \mu (T) \le \mu \bigl (\Delta _{(c/(a+b),d/(a+b))}\bigr ) + \frac{1}{a+b}. \end{aligned}$$

We now consider the two cases in the statement separately: For part (i), $c \ge a+b$ implies that $d/(a+b)\ge c/(a+b)\ge 1$. Proposition C.1 says that the first summand is $\le 1$, with equality possible only if $c=a+b$. Thus

$$\begin{aligned} \mu \bigl (\Delta _{(c/(a+b),d/(a+b))}\bigr ) + \frac{1}{a+b} \le 1+\frac{1}{2} = \frac{3}{2}, \end{aligned}$$

with equality only if $c=a+b$ and $a=b=1$.

For part (ii), $3c\ge a+b+d$ implies that $(-1/2,-1/2)$ is in $\Delta _{(c/(a+b),d/(a+b))}$, so $\Delta _{(c/(a+b),d/(a+b))}$ contains the triangle $\Delta _{(1/2,1/2)}=S(1,1,1/2)$. Its covering radius is 5/4 by Theorem 1.4. Hence,

$$\begin{aligned} \mu (T) \le \mu \bigl (\Delta _{(c/(a+b),d/(a+b))}\bigr ) + \frac{1}{a+b}\le \mu \bigl (\Delta _{(1/2,1/2)}\bigr ) + \frac{1}{a+b}\le \frac{5}{4}+\frac{1}{4}=\frac{3}{2}. \end{aligned}$$

The third inequality is strict unless $a+b=4$. Because simplices are tight, the second inequality is strict unless $\Delta _{(c/(a+b),d/(a+b))}=\Delta _{(1/2,1/2)}$, that is, unless $c=d=(a+b)/2$, which implies . $\square$

With this we can prove that all the tetrahedra in Table 1, except for the five from Lemma 3.83.8, have $\mu <3/2$:

- (1, 1, 1, 2), (1, 1, 2, 3) are the ones whose $\mu$ we computed in Corollary B.4.
- (5, 5, 5, 5) is in the conditions of part (ii) of Lemma C.3. The hypothesis that *OCD* is unimodular is trivial since $C=(0,1,0)$ and $D=(0,0,1)$.
- The four volume vectors in the third row satisfy the conditions $a+b\le c$ and $(a,b,c)\ne (1,1,2)$ of part (i) of Lemma C.3. That *OCD* is unimodular for them follows from $\gcd (a,b)=1$, because the normalized volume of *OCD* divides those of *OBCD* and *OACD*, which equal *a* and *b*, respectively.
- The four in row four satisfy the conditions $a+b\ge 4$ and $(a,b,c,d)\ne (2,2,2,2)$. The condition that *OCD* is unimodular follows again from $\gcd (a,b)=1$, except for the tetrahedron (2, 2, 3, 5).
- The remaining tetrahedron (2, 2, 3, 5) is marked “$Pyr _4(S({\mathbf {1}}_3))$” because it has a facet isomorphic to $S({\mathbf {1}}_3)$ (the facet in the plane $x+z=2y+1$) and the opposite vertex is at distance four from that facet. Lemma 2.1 applied to the projection along the base of the pyramid gives
  $$\begin{aligned} \mu (Pyr _4(S({\mathbf {1}}_3))) \le \mu (S({\mathbf {1}}_3)) + \frac{1}{4} = \frac{5}{4}. \end{aligned}$$

#### Remark C.4

All these tetrahedra except (2, 2, 2, 2) and (5, 5, 5, 5) have $\gcd (a,b,c,d)=1$. Thus, Lemmas B.1 and B.2 can be used to compute their exact covering radii, as we did for (1, 1, 1, 2), (1, 1, 2, 3) in Example B.3. The condition $a=1$ used in that computation can be weakened to $\gcd (a,V)=1$, (which these 14 tetrahedra satisfy) since then $G(V;a,b,c)=G(V;1,ba^{-1}, ca^{-1})$, where $a^{-1}$ is the inverse of *a* modulo *V*.

### The Ten Non-Tetrahedra

**Table 2:** The ten minimal non-hollow non-tetrahedra with exactly one interior lattice point, with their covering radii

$$\begin{pmatrix}1 &{} 0 &{} 0 &{} 0 &{} -1 \\ 0 &{} 1 &{} 0 &{} 0 &{} -1 \\ 0 &{} 0 &{} 1 &{} -1 &{} 0\end{pmatrix}$$	$$\begin{pmatrix}1 &{} 0 &{} 0 &{} -2 &{} -1 \\ 0 &{} 1 &{} 0 &{} -1 &{} 0 \\ 0 &{} 0 &{} 1 &{} 0 &{} -1\end{pmatrix}$$
$S({\mathbf {1}}_3) \oplus I$, $\varvec{\mu } = \mathbf{3}/\mathbf{2}$	$I\oplus Q_4$, $\mu = 4/3$
$$\begin{pmatrix}1 &{} 0 &{} -1 &{} 1 &{} -1 \\ 0 &{} 1 &{} -1 &{} 2 &{} -2 \\ 0 &{} 0 &{} 0 &{} 3 &{} -3\end{pmatrix}$$	$$\begin{pmatrix}1 &{} 0 &{} 0 &{} -2 &{} -2 \\ 0 &{} 1 &{} 0 &{} -1 &{} 0 \\ 0 &{} 0 &{} 1 &{} 0 &{} -1\end{pmatrix}$$
$Bipyr _3(S({\mathbf {1}}_3) \oplus I)$, $\mu = 17/18$	$I\oplus I\oplus I'$, $\varvec{\mu } = \mathbf{3}/\mathbf{2}$
$$\begin{pmatrix}1 &{} 0 &{} 0 &{} 0 &{} -2 \\ 0 &{} 1 &{} 0 &{} 0 &{} -1 \\ 0 &{} 0 &{} 1 &{} -1 &{} 0\end{pmatrix}$$	$$\begin{pmatrix}1 &{} 0 &{} -2 &{} 1 &{} -3 \\ 0 &{} 1 &{} -1 &{} 1 &{} -1 \\ 0 &{} 0 &{} 0 &{} 2 &{} -2\end{pmatrix}$$
$(I \oplus I')^\circ \oplus I$, $\varvec{\mu } = \mathbf{3}/\mathbf{2}$	$Bipyr _2(I\oplus I\oplus I')$, $\mu =7/8$
$$\begin{pmatrix}1 &{} 0 &{} -2 &{} 1 &{} -1 \\ 0 &{} 1 &{} -1 &{} 1 &{} -1 \\ 0 &{} 0 &{} 0 &{} 2 &{} -2\end{pmatrix}$$	$$\begin{pmatrix}1 &{} 0 &{} 0 &{} -1 &{} 0 &{} 0 \\ 0 &{} 1 &{} 0 &{} 0 &{} -1 &{} 0\\ 0 &{} 0 &{} 1 &{} 0 &{} 0 &{} -1\end{pmatrix}$$
$Bipyr _2((I \oplus I')^\circ \oplus I)$, $\mu = 1$	$I\oplus I\oplus I$, $\varvec{\mu } = \mathbf{3}/\mathbf{2}$
$$\begin{pmatrix}1 &{} 0 &{} 0 &{} -1 &{} 1 \\ 0 &{} 1 &{} 0 &{} -1 &{} 1 \\ 0 &{} 0 &{} 1 &{} 0 &{} -1\end{pmatrix}$$	$$\begin{pmatrix}1 &{} 0 &{} -1 &{} 0 &{} 1 &{} -1 \\ 0 &{} 1 &{} 0 &{} -1 &{} 1 &{} -1 \\ 0 &{} 0 &{} 0 &{} 0 &{} 2 &{} -2\end{pmatrix}$$
$$ Pyr _3([0,1]^2)$$, $$\mu = 4/3$$	$$Bipyr _2(I\oplus I\oplus I)$$, $$\mu = 3/4$$

For the ten polytopes in Table 2 we use the following direct arguments:

- Four of them are the non-tetrahedra in Lemma 3.83.8, of covering radius 3/2.
- There are another four that are affinely equivalent to the previous four, except considered with respect to a finer lattice. They are marked as $Bipyr _i(\,{\cdot }\,)$, where *i* is the index of the superlattice, since they are also (skew) bipyramids over their intersection with the plane $z=0$. This intersection is, in the four cases, one of the three non-hollow lattice polygons with $\mu =1$. Lemma 2.1 for the projection $\pi$ onto the *z*-coordinate gives
  $$\begin{aligned} \mu (P) \le \mu (P\cap \{z=0\}) + \mu (\pi (P)) \le 1 + \frac{1}{4} =\frac{5}{4}, \end{aligned}$$
  since $\pi (P)$ has length at least four in all four cases.
- The one marked $Pyr _3([0,1]^2)$ is a pyramid with base a unimodular parallelogram in the plane $x+y+z=1$ and apex at distance three. Lemma 2.1 applied to the projection along the base gives
  $$\begin{aligned} \mu (Pyr _3([0,1]^2)) \le 1 + \frac{1}{3}. \end{aligned}$$
- The remaining one is marked $I\oplus Q_4$ because it decomposes as
  $$\begin{aligned} \begin{pmatrix}0 &{} -2 \\ 1 &{} -1 \\ 0 &{} 0\end{pmatrix}\oplus \begin{pmatrix}1 &{}-1 &{} 0 &{} -1 \\ 0 &{} 0 &{} 0 &{} 0 \\ 0 &{} 0 &{} 1 &{} -1\end{pmatrix}, \end{aligned}$$
  where the first summand is equivalent to $I=[-1,1]$ and the second is a quadrilateral $Q_4$. Since $Q_4$ strictly contains a translation of $S({\mathbf {1}}_3)$ and $S({\mathbf {1}}_3)$ is tight (Lemma 2.7), we have
  $$\begin{aligned} \mu (I\oplus Q_4) = \mu (I)+ \mu (Q_4) < \mu (I)+ \mu (S({\mathbf {1}}_3)) = \frac{3}{2}. \end{aligned}$$

## Footnotes

1 The notation $S({\mathbf {1}}_{d+1})$ comes from the fact that this is a particular case of the simplices $S(\omega )$, $\omega \in {\mathbb {R}}_{>0}^{d+1}$, introduced below. We call $S({\mathbf {1}}_{d+1})$ the *standard terminal simplex* since *terminal* is used in the literature for lattice simplices with the origin in the interior and no lattice points other than the origin and the vertices.

## References

[1] Averkov, G., Basu, A.: Lifting properties of maximal lattice-free polyhedra. Math. Program. **154**(1–2), Ser. B, 81–111 (2015)

[2] Balletti, G., Kasprzyk, A.M.: Three-dimensional lattice polytopes with two interior lattice points (2016). arXiv:1612.08918

[3] Beck, M., Sanyal, R.: Combinatorial Reciprocity Theorems. An Invitation to Enumerative Geometric Combinatorics. Graduate Studies in Mathematics, vol. 195. American Mathematical Society, Providence (2018)

[4] Bey C, Henk M, Wills JM (2007). Notes on the roots of Ehrhart polynomials. Discrete Comput. Geom..

[5] Blanco M, Santos F (2019). Non-spanning lattice $$3$$-polytopes. J. Combin. Theory Ser. A.

[6] Castryck W (2012). Moving out the edges of a lattice polygon. Discrete Comput. Geom..

[7] Codenotti, G., Santos, F., Schymura, M.: The covering radius and a discrete surface area for non-hollow simplices (2019). arXiv:1903.02866v1 10.1007/s00454-021-00330-3 PMC8709830 35023883

[8] Cook W, Koch T, Steffy DE, Wolter K (2013). A hybrid branch-and-bound approach for exact rational mixed-integer programming. Math. Program. Comput..

[9] Ewald, G.: Combinatorial Convexity and Algebraic Geometry. Graduate Texts in Mathematics, vol. 168. Springer, New York (1996)

[10] González Merino B, Schymura M (2017). On densities of lattice arrangements intersecting every $$i$$-dimensional affine subspace. Discrete Comput. Geom..

[11] Gruber, P.M.: Convex and Discrete Geometry. Grundlehren der Mathematischen Wissenschaften, vol. 336. Springer, Berlin (2007)

[12] Gruber, P.M., Lekkerkerker, C.G.: Geometry of Numbers. North-Holland Mathematical Library, vol. 37. North-Holland, Amsterdam (1987)

[13] Hadwiger H (1970). Volumen und Oberfläche eines Eikörpers, der keine Gitterpunkte überdeckt. Math. Z..

[14] Henze M, Malikiosis R-D (2017). On the covering radius of lattice zonotopes and its relation to view-obstructions and the lonely runner conjecture. Aequat. Math..

[15] Iglesias-Valiño Ó, Santos F (2019). Classification of empty lattice $$4$$-simplices of width larger than two. Trans. Am. Math. Soc..

[16] Kannan R (1992). Lattice translates of a polytope and the Frobenius problem. Combinatorica.

[17] Kannan R, Lovász L (1988). Covering minima and lattice-point-free convex bodies. Ann. Math..

[18] Kasprzyk AM (2010). Canonical toric Fano threefolds. Can. J. Math..

[19] Lenstra HW (1983). Integer programming with a fixed number of variables. Math. Oper. Res..

[20] Lovász, L.: Geometry of numbers and integer programming. In: Mathematical Programming (Tokyo 1988). Math. Appl. (Japanese Series), vol. 6, pp. 177–201. SCIPRESS, Tokyo (1989)

[21] Marklof J, Strömbergsson A (2013). Diameters of random circulant graphs. Combinatorica.

[22] Martinet, J.: Perfect Lattices in Euclidean Spaces. Grundlehren der Mathematischen Wissenschaften, vol. 327. Springer, Berlin (2003)

[23] Paat, J., Weismantel, R., Weltge, S.: Distances between optimal solutions of mixed-integer programs. Math. Program. **179**(1–2), Ser. A, 455–468 (2020)

[24] Schnell U (1992). Minimal determinants and lattice inequalities. Bull. Lond. Math. Soc..

[25] Schnell U (1995). A Minkowski-type theorem for covering minima in the plane. Geom. Dedicata.

